# Fluctuating Finite Element Analysis (FFEA): A continuum mechanics software tool for mesoscale simulation of biomolecules

**DOI:** 10.1371/journal.pcbi.1005897

**Published:** 2018-03-23

**Authors:** Albert Solernou, Benjamin S. Hanson, Robin A. Richardson, Robert Welch, Daniel J. Read, Oliver G. Harlen, Sarah A. Harris

**Affiliations:** 1 School of Physics and Astronomy, University of Leeds, Leeds, United Kingdom; 2 School of Chemistry, University College London, London, United Kingdom; 3 School of Mathematics, University of Leeds, Leeds, United Kingdom; 4 Astbury Centre for Structural and Molecular Biology, University of Leeds, Leeds, United Kingdom; Universite de Montreal, CANADA

## Abstract

Fluctuating Finite Element Analysis (FFEA) is a software package designed to perform continuum mechanics simulations of proteins and other globular macromolecules. It combines conventional finite element methods with stochastic thermal noise, and is appropriate for simulations of large proteins and protein complexes at the mesoscale (length-scales in the range of 5 nm to 1 μm), where there is currently a paucity of modelling tools. It requires 3D volumetric information as input, which can be low resolution structural information such as cryo-electron tomography (cryo-ET) maps or much higher resolution atomistic co-ordinates from which volumetric information can be extracted. In this article we introduce our open source software package for performing FFEA simulations which we have released under a GPLv3 license. The software package includes a C ++ implementation of FFEA, together with tools to assist the user to set up the system from Electron Microscopy Data Bank (EMDB) or Protein Data Bank (PDB) data files. We also provide a PyMOL plugin to perform basic visualisation and additional Python tools for the analysis of FFEA simulation trajectories. This manuscript provides a basic background to the FFEA method, describing the implementation of the core mechanical model and how intermolecular interactions and the solvent environment are included within this framework. We provide prospective FFEA users with a practical overview of how to set up an FFEA simulation with reference to our publicly available online tutorials and manuals that accompany this first release of the package.

This is a *PLOS Computational Biology* Software paper.

## Introduction

The enormous complexity of biomolecules and their interactions means that molecular modelling and simulation have proven invaluable for interpreting experimental data and providing physical insight into biomolecular mechanisms [1]. At the atomistic and near-atomistic level, molecular dynamics (MD) simulations have been widely used to complement experimental structural data, and to include dynamical effects (such as thermal fluctuations) that are known to be important to function, but which are inaccessible experimentally. Computational advances over the past few decades have taken us from all-atom simulations of small molecules such as bovine pancreatic trypsin inhibitor, with only 58 residues [2], to simulations of huge macromolecular systems such as the HIV-1 capsid [3], containing a staggering 64 million atoms in total. The new field of structural systems biology has enabled comprehensive 3D models of cell-scale structures to be constructed in molecular [4]and atomistic detail [5] for future simulations. High-resolution simulations of these systems will allow computational biophysicists to understand how nanoscale structure and dynamics give rise to biological function, effectively a ‘computational microscope’ into the biological world [6].

At large length-scales, from the micron upwards, a representation based on continuum mechanics is often a more suitable and efficient way to describe the evolution of a system. Methods built on continuum fluid mechanics such as Lattice-Boltzmann can be used for simulations of the flow of biological fluids [7], and finite element methods are routinely employed for solid tissue modelling [8, 9].

Mesoscopic processes that occur over time-scales of milliseconds to seconds and length-scales of between 10 nm and 1 μm, such as the walking of cytoplasmic dynein along microtubules [10], the aggregation of fibrin molecules during blood clotting [11], and the assembly of the kinetochore during cell division [12], are also crucial to biological function. For these intermediate mesoscopic length-scales and time-scales there are fewer computational tools available. Coarse-graining strategies to access larger length and time-scales than atomistic techniques have most commonly been “bottom-up”. Single atoms are clustered into larger beads, and interactions are described by potentials of mean force which may either be knowledge-based or derived from physical principles [13–15]. Most of the commonly used coarse-grained models are relatively high resolution, and thus only provide modest increases in accessible system sizes. Highly coarse grained methods, such as Dissipative Particle Dynamics [16] or Ultra-Coarse-Graining theory [17] are also active areas of development. The latter provides a physical rationale for systematically clustering large parts of a biomolecule (such as an individual protein domain) into a single bead, that is itself able to reside in different internal meta-states. Similarly, particle-based reaction-diffusion dynamics [18, 19] has been used as a simplified model to study the diffusion, reaction and self-assembly of biomolecules in cellular environments.

Other techniques allowing access to the mesoscale involve treating macromolecules as rigid bodies. These models have provided vital information about protein diffusion [20], cytoplasmic crowding [5, 21], and pathways for the assembly of virus capsids [22], but their method of coarse-graining discounts the effects of protein deformation on diffusional dynamics, so cannot be used for highly dynamic biomolecules such as molecular motors. Until recently, there was also a paucity of experimental structural information available for biomolecular complexes at the mesoscale, due to the technical challenges involved in preserving delicate macromolecular complexes in their intact, native states. However, recent advances in biophysical tools such as cryo-electron microscopy and tomography [23] and super-resolution microscopy are now starting to generate a wealth of structural and dynamic information at precisely this length-scale. Integrative modelling [24] is an effort to characterise large macromolecular assemblies by combining complementary experimental information from multiple sources, but it does not provide a means to study protein mechanics.

In response, we have developed a new software tool “Fluctuating Finite Element Analysis” (FFEA) which uses a continuum representation of biomolecules, thereby modelling the mesoscale “top down”, rather than “bottom up”. It represents the volumetric shape of proteins using a 3D tetrahedral finite element mesh to which we apply thermal fluctuations, providing a dynamic trajectory showing how the protein changes shape over time due to its own structure and in response to its interactions with other molecules [25]. The initial co-ordinates for constructing the finite element mesh can come directly from 3D volumetric data, such as cryo-electron tomography maps, or from atomistic structures following an appropriate effective surface calculation method. Algorithms for calculating atomic surfaces based on solvent accessibility or electrostatic repulsion are readily available, implemented within existing visualisation software such as Chimera [26] or VMD [27].

FFEA uses a viscoelastic constitutive model to represent the continuous deformation of the enclosed volume. This model describes the protein mechanics in such a way that the biomolecule will always relax back towards its equilibrium shape following an external perturbation in a dissipative manner, rather than partially retaining induced deformation via creep effects. The protein models are parameterised by assigning continuum material parameters to the finite element mesh (presently two elastic moduli and two dynamic internal viscosities, though more complicated constitutive models are possible), which define how compliant and dissipative the macromolecule will be when subject to thermally induced deformation. Protein-protein interactions are modelled by a volume exclusion term, preventing different biomolecules occupying the same space within a simulation, together with a short range attractive term, the magnitude and range of which are chosen by the user to best mimic the strength of the specific interaction of interest.

FFEA is designed to operate over length-scales of between 5 nm and 1 μm, which encompasses large macromolecular and subcellular structures such as the nuclear pore complex, the kinetochore, the sarcomere, the axoneme and the cytoskeleton, for example. At this higher level of biomolecular structure and organisation, it is potentially more instructive to consider whole proteins, rather than atoms or molecular subunits, as comprising the fundamental irreducible unit in the simulation. At this scale the proteins are soft nanoscale objects that operate in an environment dominated by thermal noise. Additionally, FFEA has no upper length-scale as, in systems that are sufficiently large, the thermal effects become negligible and FFEA naturally reduces to conventional finite element analysis. At this upper limit, however, well established finite element analysis software can, and should, be used in its place to eliminate the now superfluous calculations of thermal fluctuations. Conversely, since the continuum approximation breaks down when elements comprising the mesh become smaller than ∼5 Å, there is a fundamental limit on the resolution of the method, below which coarse-grained or atomistic MD is more appropriate.

The physical properties of an FFEA protein model share some similarities with Gaussian or Elastic Networks [28], which use a network of beads and harmonic springs to represent the structure and dynamics of proteins, and which therefore capture approximately the elastic component of the FFEA viscoelastic constitutive model. This similarity between conventional finite element analysis and network models has been studied previously [29]. However, while many (although not all) Gaussian/Elastic Network models only include unbreakable harmonic interactions to simplify the solution of the equations of motion, within FFEA we can represent non-bonded interactions between and within individual proteins within a complex. Moreover, in FFEA the volumetric space within each finite element is filled with material, while in particle-spring models it remains empty. For very large macromolecules, especially those containing irregular shapes such as very long coiled-coil regions, it can be difficult to ensure that beads are sufficiently closely spaced within a Gaussian/Elastic network model to maintain the shape of the complex and to prevent steric overlap between the different proteins in the simulation. Continuum FFEA models also naturally include torsional rigidity, which can be particularly important to the dynamics of irregular and non-spherical proteins, and indeed such differences with Elastic Network Models have been shown in the case of Vacuolar-type ATPases [30].

In this paper we present an open source software package that implements FFEA. The package is licensed under GPLv3 and can be downloaded from https://bitbucket.org/ffea/ffea. The paper begins with a description of the internal mechanics involved in FFEA and our treatment of protein-protein interactions. We then describe how the resulting algorithms have been implemented within the FFEA code base and the overall structure of the software package, along with the accompanying visualisation and analysis tools, user tutorials, documentation and test suite. Next, we demonstrate the use of FFEA in modelling the molecular chaperone GroEL. Finally, we discuss the future directions for FFEA and for computational exploration of the biomolecular mesoscale more generally. For readers interested in the mathematical background to this work, further detail is provided as supplementary material in the S1 File.

In the following sections we describe how the protein mechanics are represented within FFEA, and explain how the effect of the solvent environment, protein-protein interactions and structural restraints have been modelled.

### Physics of FFEA

FFEA describes the time evolution of a system of *N* interacting viscoelastic bodies subject to thermal fluctuations. In viscoelastic bodies, the mechanical response to stress depends not only on the strain (as in purely elastic bodies) but also on the strain rate (as in viscous fluids). We model viscoelasticity through the Kelvin-Voigt constitutive model, allowing the viscous and elastic stresses to be calculated separately. This is one of the simplest models that replicates the stress response of viscoelastic solids. Therefore, we write the equation of motion (Cauchy’s momentum equation):

$$\rho \frac{Du}{Dt}=\nabla \cdot \left(\sigma^{v}+\sigma^{e}+\pi \right)+f$$ (1)

where *ρ* is the density, *D**u***/*Dt* the material derivative of the velocity with respect to time, ***σ***^*v*^ the viscous stress, ***σ***^*e*^ the elastic stress and ***f*** is the external force density. Additionally, we include stochastic thermal noise as an additional component of stress, ***π***. A detailed mathematical description of these terms can be found in the S1 File.

Within the Kelvin-Voigt model, the elastic stress can be independently derived from a strain energy density functional. We chose a constitutive model able to model compressibility, which in our case consists of a hyperelastic response to shear deformation combined with an isotropic resistance to changes in volume. This requires that the user specifies both bulk and shear moduli for the biomaterial. Different sections of a biomolecule may be assigned different values for these moduli.

The internal viscosity of the biomolecule itself arises from an internal friction and therefore has an associated energy loss. At finite temperature, this viscosity is fundamentally coupled to the statistical distribution of the stochastic thermal noise experienced by the molecule (as discussed in more detail below). We model the viscous stress as that of a Newtonian fluid, linear with respect to the strain rate. This requires that the user specifies both bulk and shear viscosities for the biomaterial, which again may be inhomogeneous throughout the volume.

In addition to the internal viscosity, we also define an external viscosity component resulting from interactions with the solvent in which the biomolecule is immersed. We include this effect by assigning an additional local viscous drag force to each node that is proportional to its own velocity relative to a fixed background, together with a corresponding fluctuating thermal force with statistics that satisfy the fluctuation-dissipation theorem (this treatment is equivalent to standard Langevin Dynamics). The local nature of the solvent interaction means that there is presently no inter-molecular hydrodynamic coupling between biomolecules, as there are no correlations between the frictional and thermal forces between nodes in different molecules. Within a single molecule, some hydrodynamic coupling is mediated through the internal viscosity, but not through the external solvent. The importance of hydrodynamic effects in biomolecular systems has been extensively debated [31], and a possible treatment, if necessary, could be implemented in the future by coupling the finite element mesh to the solvent using a suitable boundary element method.

#### Thermal noise within the protein and the effect of the external solvent

From Eq 1, ***π*** is the total stochastic thermal stress which provides energy to the protein model in the form of thermal fluctuations. The values of this term are therefore random, but statistically distributed to fulfill the fluctuation-dissipation theorem so that at equilibrium, the kinetic and strain energies of each degree of freedom are correctly distributed. Contributions to ***π*** come from both internal thermal fluctuations, arising from a coupling to the internal viscosity of the biomolecule, as well as the external thermal fluctuations of the solvent which are coupled to the external solvent viscosity.

Following an approach equivalent to that used by Sharma and Patankar [32], our stress tensor is *δ*-correlated in both time and space, and since our viscous dissipation depends only upon the instantaneous deformation rate, this approach allows the fluctuating stress to be calculated entirely locally. It follows that each contribution to the thermal stress, whether arising from internal or external viscosities, is statistically independent and can be individually coupled to their respective viscosities to ensure the correct thermodynamic behaviour. In FFEA, the user has control as to whether to include thermal fluctuations to the simulation or not, and can also choose whether or not to include an external solvent. Input parameters for the temperature and the external viscosity are read from the input file.

#### Discretisation and protein dynamics

We employ the finite element method to solve Eq 1 in the general case by discretizing the material into simple geometric elements. We use tetrahedral elements in which the material velocity is linearly interpolated between the values at the vertices of the tetrahedra, which are also called *nodes* within the finite element framework. Whilst other element geometries are possible, for example hexahedral bricks, tetrahedra are preferable for two reasons. First, it is always possible to create a tetrahedral mesh from a set of points in a body. Second, this provides an implementation for the thermal noise which is local to each element.

The resulting discretised equation of motion is given by:

$$\begin{array}{c} M_{pq}\frac{dv_{q}}{dt}=-\Lambda_{pq}v_{q}+E_{p}+N_{p}+O_{p} \end{array}$$ (2)

where the summation convention implies a sum over the index *q*. The indices *p* and *q* correspond to the three spatial coordinates for each node in the mesh. Here, *v*_*q*_ is a component of velocity, *M*_*pq*_ is the mass matrix which distributes the density contained within an element to its associated nodes, and Λ_*pq*_ the viscosity matrix resulting from internal and external viscosities. *E*_*p*_ is the elastic force vector which, although conservative, is a non-linear function of node position. Finally, *N*_*p*_ is the stochastic noise force vector, and *O*_*p*_ represents all additional conservative external forces. By following the trajectory of each node we can determine the volumetric deformation of each element. This captures the continuum nature of the protein and is therefore fundamentally different from standard bead-spring models. The contents of the mass matrix, *M*_*pq*_, for example depends on how the *density* of the material varies throughout the biomolecule, rather than assigning a mass explicitly to each node. In our own simulations we have generally assumed the density to be homogeneous, but our software permits material inhomogeneity as well.

#### Temporal discretisation

In order to solve Eq 2 we must also introduce a numerical integration scheme. The simplest scheme to use is a forward Euler scheme in which the time derivative is replaced by the finite difference: [*v*_*q*_(*t* + Δ*t*) − *v*_*q*_(*t*)]/Δ*t* for a time-step Δ*t*. This first-order scheme was found to give an acceptable trade-off between accuracy and computational speed in the original implementation of FFEA [25]. Thermal equilibration tests verifying this are included in the FFEA test suite, and are discussed later.

However, in cases where the mass is small, this requires very small time-steps for stability. As discussed in [33], for many biomolecular applications, the time-scale over which the mass affects the dynamics is often small compared to the time-scale of interest, and in these cases it is more efficient to seek an implicit solution in which we assume, as in the case of Brownian dynamics, that the motion relaxes rapidly to the velocity at which the forces are in equilibrium. By seeking the solution of Eq 2 for which the LHS is zero, we obtain:

$$\begin{array}{c} \Lambda_{pq}v_{q}=E_{p}+N_{p}+O_{p} \end{array}$$ (3)

From here on, Eqs 2 and 3 will be referred to later in the text respectively as the Langevin and Brownian equations for FFEA. The software allows the user to choose either equation of motion for each individual biomolecule involved in the system.

### Protein-protein interactions within FFEA

The interactions between different viscoelastic biological bodies, or *blobs* (BioLOgical BodieS), are taken into account through the body forces in ***f***, which transforms into the vector *O*_*p*_ following the application of the finite element method.

#### Steric repulsion within FFEA

**Fig 1:**
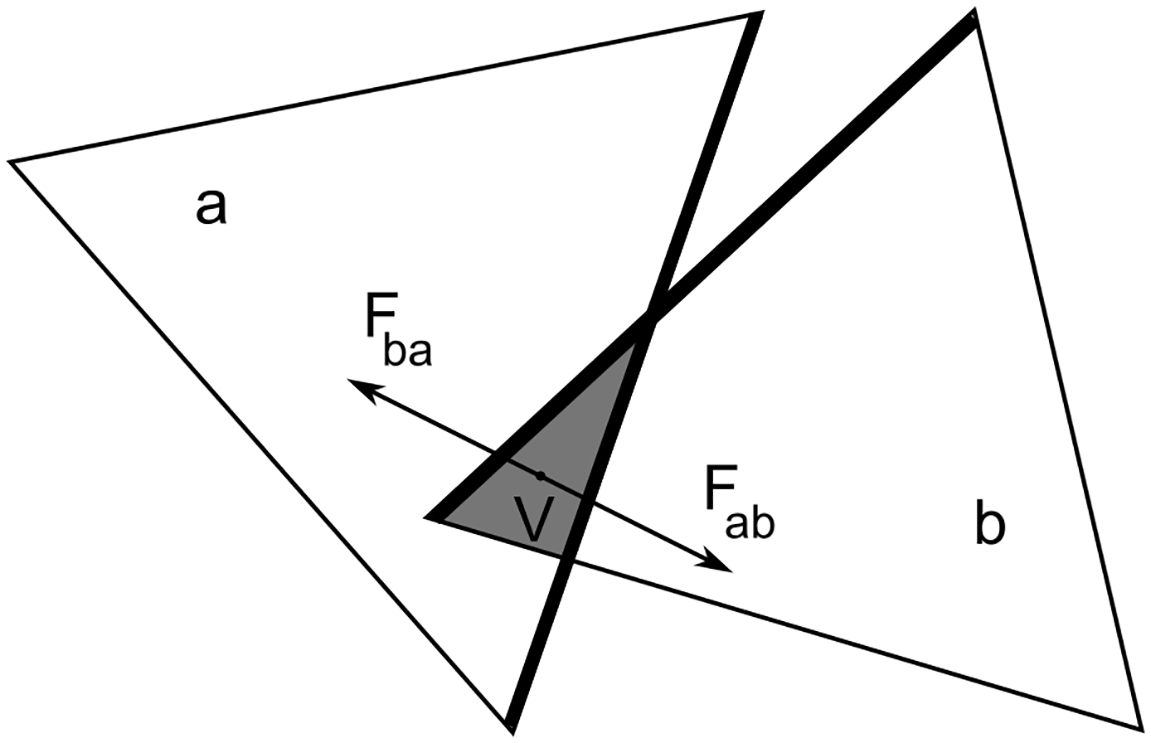
2D illustration of the steric repulsion implemented in FFEA, where 3D tetrahedra have been reduced to triangles. The intersecting elements *a* and *b* gain a positive energy proportional to the area enclosed by their intersection, *V* (labelled *V* as it is a volume intersection in 3D). The repulsive force resulting from the negative spatial gradient of *V* is applied at the centre of the enclosed volume, and interpolated linearly onto the nodes of the two involved elements.

We have introduced a soft potential energy term specifically to describe steric repulsion. In this scheme, interacting *blobs* gain an unfavourable positive energy that is proportional to their intersecting volume. Since this is path-independent, the resulting repulsive force is conservative. Furthermore the total overlapping volume, *V*, is equal to the sum of the overlapping tetrahedra, and therefore the calculation of this energy is independent of how the system is partitioned, facilitating algorithm parallelisation. The resulting repulsive force between two tetrahedra is calculated as the numerical gradient of this intersecting volume ***F***_*steric*_ = −∇*V*, and applied at the centre of the overlapping volume elements. We then use the finite element method to linearly interpolate this onto the nodes of the interacting elements (see Fig 1). To lessen the computational load as much as possible, this steric overlap term is only computed for tetrahedra at the blob surface, the list of which is calculated at the beginning of the simulation. Implementing the steric overlap term requires FFEA to identify all interacting face pairs from this list, which can be quickly achieved using an algorithm developed by Ganovelli *et al.* [34]. The intersecting volume is then efficiently calculated using the method of Franklin and Kankanhalli [35].

#### Attractive interactions within FFEA

In particle-based atomistic and coarse-grained simulations, Lennard-Jones (LJ) interactions are commonly used to represent short range attractive forces and to suppress steric overlap. We have implemented an equivalent potential function in the continuum regime within FFEA using surface-surface interactions. The force per unit area exerted at a point ***s*** on the surface Γ_*s*_ due to another surface Γ_*t*_ can be written as:

$$\begin{array}{c} F\left(s\right)=\int_{\Gamma_{t}}f\left(s,t\right)dA_{t} \end{array}$$ (4)

where ***t*** is a point on the surface Γ_*t*_, and ***f***(***s***, ***t***) is a force per unit area at point ***s*** per unit area at ***t*** with the LJ form:

$$\begin{array}{c} f\left(s,t\right)=\frac{12\epsilon }{r^{eq}}\left[{\left(\frac{r^{eq}}{r\left(s,t\right)}\right)}^{13}-{\left(\frac{r^{eq}}{r\left(s,t\right)}\right)}^{7}\right]\hat{r} \end{array}$$ (5)

where *r*(***s***, ***t***) is the distance between any points ***s*** and ***t***, *r*^*eq*^ is the equilibrium distance for this interaction and *ϵ* is the energy minimum occurring at *r*(***s***, ***t***) = *r*^*eq*^. Discretising the volume of the interacting biomolecules into tetrahedra has the effect of partitioning the surfaces into sets of triangles, or faces. Including these LJ interactions results in attracting but impenetrable patches that when discretised into surface-surface interactions are computed through the double sum over all faces in the system. A detailed description on how this is computed within FFEA is provided in the S1 File.

While the Lennard Jones potential provides a convenient method for describing mid-range attractive and short-range repulsive interactions using a single potential, the hard-core nature of the repulsive potential introduces computational difficulties, as a very short time-step is required to avoid energy escalation from inter-penetration of proteins during the simulation. This can be solved by employing a combination of the steric and Lennard-Jones potentials, where the former accounts for the hard-core repulsion and the latter for the attraction. To transition between the two regimes when 0 < *r*(***s***, ***t***) < *r*^*eq*^, we use an analytical polynomial interpolation designed to be continuous in both force and energy.

#### Precomputed potentials in FFEA

**Fig 2:**
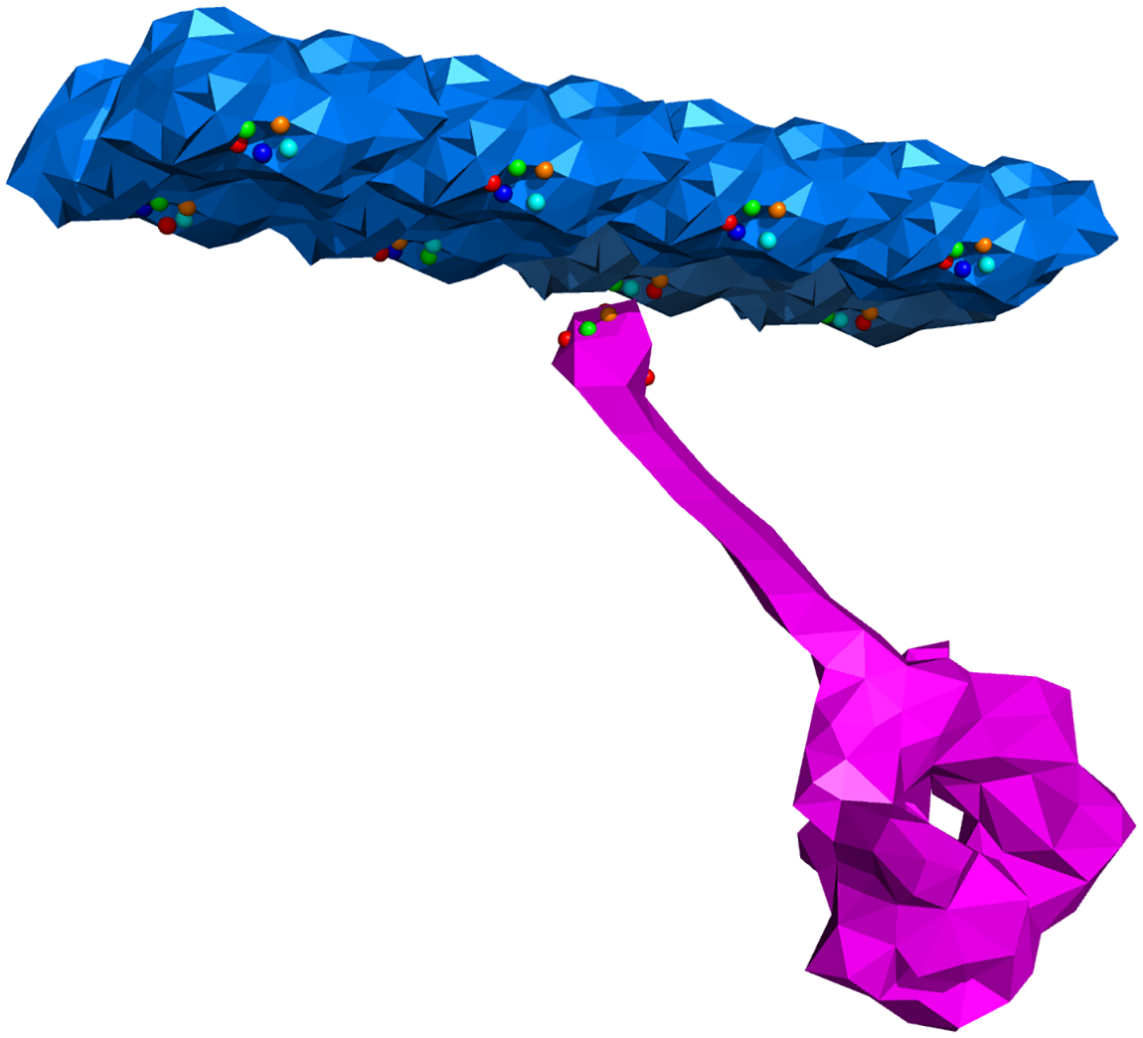
Five different bead types (shown in different colours) control the interactions of the stalk of the dynein molecular motor (pink) and the microtubule track (blue). Each bead is assigned to an element of the mesh, and the forces resulting from the tabulated interaction potentials between beads are interpolated linearly onto the nodes of the elements, thus transmitting the force to the corresponding body.

To improve the modelling flexibility within FFEA, we have implemented the ability to include precomputed tabular potentials as external forces. This is a common approach in molecular modelling packages, such as Gromacs [36] or NAMD [37], allowing FFEA to incorporate specific interactions between functional elements. For situations where experimental information for biomolecular affinities are unavailable, these can be obtained by coarse-graining from atomistic MD simulations. This procedure provides sets of pair-wise interaction forces as a function of the separations of predefined ‘pseudo-particles’ which are considered to be the interacting units during coarse-graining. Once these potentials have been defined, interacting beads are embedded within the volumes of the finite element meshes (see Fig 2), so that each pseudo-particle bead is permanently associated with the tetrahedra having the closest centroid in the initial configuration of the FE mesh. The resulting force calculated from the gradient of this potential is then linearly interpolated throughout the corresponding elements to the nodes, again using the finite element shape functions, and included in the force vector *O*_*p*_.

### Harmonic restraints and frozen nodes within FFEA

It is often useful to include harmonic restraints within biomolecular simulations, either to maintain a particular biomolecular configuration, or to steer an existing conformation into a new state. In FFEA, harmonic restraints can be imposed using Hookean springs to form a simple link between pairs of nodes. The equilibrium distance and the force constant of the Hookean potential can be defined by the user.

The utility to completely freeze the position of either an entire macromolecule, or a selected subset of nodes within a macromolecule, over the course of a simulation is also available. These rigid restraints could be used, for example, to simulate large structures such as microtubules. These superstructures do not deform significantly compared to smaller, more flexible proteins, yet they still interact sterically and through short-range attractive forces, such as at specific binding sites. Rigid restraints reduce the computational load and simplify calculations for simulations involving these kinds of structures.

## Design and implementation

The FFEA software package is divided into two parts. The “FFEA Runner” is a C ++ implementation of the FFEA algorithms, which computes trajectories containing information on how the thermally fluctuating viscoelastic objects within the simulation change shape and interact with one another. “FFEA Tools” are provided for users to set up the FFEA simulations themselves and to analyse the resulting trajectories. These tools are composed of a Python package with a full Application Programming Interface (API), a viewer implemented as a plugin for PyMOL, and a collection of Python analysis tools under a single command interface.

Setting up a system and performing an FFEA simulation is accomplished via a command line interface. We have provided detailed documentation accompanying the package that can be either read directly from the text files, or built using Doxygen [38], a documentation generating tool, and viewed in a web browser. The whole FFEA package, including the documentation, can be freely downloaded and easily configured on any UNIX platform using CMake, while the Python package within the FFEA tools can be installed on its own using Python setuptools. We have also successfully built the software using the Windows 10 subsystem for Linux. Furthermore, compiled packages for Linux x86 machines are also provided.

### FFEA runner

#### General overview

The overall structure of the FFEA code is analogous to a conventional molecular dynamics program. Following an initialisation phase, a loop of *n*_*t*_ time-steps is performed, within each of which the program determines all forces affecting each body and subsequently integrates the equations of motion to move the system forward in time. Every *n*_*c*_ steps the program outputs the details of the simulation (positions and all quantities required for restart, together with the configurational energy, centre of masses, RMSD, etc).

Within each time-step, the calculation of the forces is deterministic except for the thermal force. Since it is a stochastic function, the thermal force requires a suitable pseudo-random number generator (RNG) to calculate reliably each value and retain the correct statistical properties throughout the simulations. The FFEA software package uses, and is distributed with, an implementation of the random number generator (RNG) RngStreams [39, 40]. This provides a large number of long, uncorrelated streams (period 2^127^), and has the ability to save the state of each active stream at any point, which is essential to stopping and restarting FFEA calculations while avoiding any risk of having correlated random numbers [41]. Adaptations of RngStreams are used in a number of software packages such as Arena, Automod, Inosim, Matlab, R, SAS, and others.

FFEA is parallelised using OpenMP, and two executables are produced by compiling the code using different compiler flags automatically through through CMake (ffea and ffea_mb). Under the first scheme (ffea), several loops running over the number of elements and nodes are parallelised, while under the second scheme (ffea_mb) loops running over the different bodies are parallelised. The “loop over bodies” scheme performs better in systems where multiple *blobs* are simulated, as it includes fewer synchronisation points, while the “loop over elements” scheme is appropriate for cases where a single large *blob* is simulated. For each initialised thread in either scheme, RngStreams is able to provide a separate RNG stream local to that thread. Each stream remains uncorrelated from all others as well as itself over the course of the simulation. This increases the computational robustness of the scheme, with a view to future development of the parallelisation procedures.

#### Face-face interactions

Within the code structure, the Lennard-Jones and steric interactions are implemented as interactions between *faces*, not *nodes*. As it is a softer potential, steric repulsion is the recommended option to keep molecules from passing through one another, whether combined with Lennard-Jones interactions or not. For a system with *N*_*f*_ interacting faces in total, the total number of face-face interactions to be calculated in a given time-step can be reduced from $N_{f}^{2}$ to a much lower number of calculations using linked-lists [42]. In this algorithm, the simulation box is tiled into tessellating cuboidal cells, or *voxels*, and each potentially interacting face is assigned to the voxel where it has its centroid, an *O*(*N*_*f*_) operation. At every time-step, face-face interactions are calculated only for pairs assigned to the same or adjacent voxels, significantly reducing the computational cost of the face-face interactions, which are usually $O(N_{f}^{2})$, whilst retaining physical accuracy. Updating this linked-list structure is performed every *n*_*nl*_ time-steps as a background operation, using a specific task thread, and both the size of the voxels and *n*_*nl*_ can be controlled from the input file. The voxel size should ideally be slightly larger than the interaction range, which corresponds to the desired cutoff on the Lennard-Jones potential, or roughly half of the length of the largest edge in the steric potential.

Further reduction in computational cost comes from only considering faces pointing towards each other i.e., ***n***_1_ · ***n***_2_ < 0 (where ***n***_*i*_ is the normal vector to the face *i*) which avoids transmitting the interaction through the protein interior as well as acting as a fast numerical check before computing the full face-face interaction.

In addition to the parameters related to the linked-lists, users can assign a ‘type’ to each of the faces. Each type, *t*, is simply an integer such that −1 ≤ *t* < *N*_*t*_. In the current implementation, *N*_*t*_ = 7 but can be increased if needed. *t* = −1 represents an inactive face, which will not interact with anything, and the remaining indices represent different active types. Therefore, for each interacting face pair there exists an associated type pair, {*t*, *s*}. Steric interactions only check whether a face is active or inactive before performing a calculation, whereas LJ interactions consider the different types. For each possible pair interaction type pair, the user is able to provide different associated LJ parameters $r_{ts}^{eq}$ and *ϵ*_*ts*_ for that interaction type. Thus, FFEA allows a large number of different LJ type interactions within the same simulation.

#### Pre-computed potentials

The set up procedure when using pre-computed potentials associates each bead with the element having the closest centroid. The user has the freedom to restrict this global search to a provided range of elements. The viewer can be of help when finding the relevant element indices.

The tabulated potential is then read in from external files, one for each bead-pair type. If a file is not found for a certain type of bead-pair, the interaction is considered null. At every time-step, the absolute position for every bead is calculated together with the energies and the forces, using the same linked lists algorithm and associated background update used in face-face interactions. The resulting dynamics are implemented by interpolating the forces linearly onto the nodes of the corresponding elements.

#### Linear elastic model and time-scale calculator

Before performing a full non-linear simulation of the stochastic equations Eqs 2 or 3, we have found it is convenient to obtain an initial and rapid appreciation of the expected modes and range of motion of an individual molecule due to thermal fluctuation together with an estimate of typical time-scales for the motion. This is possible via a linear approximation to the elastic force vector in Eqs 2 or 3. We have integrated additional tools in the main FFEA code to implement this process: these are, the “linear elastic model” (LEM) and “time-scale calculator”.

The main equations of motion Eqs 2 and 3 both contain the elastic force vector, ***E***, which is a non-linear function of deformation. A linear expansion of this vector with respect to the initial node positions generates a spring constant matrix, *K*_*pq*_, which defines the effective linear elastic constants between each pair of nodes. Diagonalisation of this matrix gives a set of eigenvectors corresponding to the normal modes of motion of the system, and the associated eigenvalues the relative stiffness of each mode. The user can output this both as raw data and in the form of FFEA trajectories, the collection of which we term the FFEA linear elastic model.

In addition, suitable combinations of both the mass matrix and the spring constant matrix with the viscosity matrix give two matrices containing approximate time-constants, *τ*_*pq*_, for the system. Diagonalisation of these matrices will generate the full spectrum of time-scales within the system, allowing the user to make a more educated choice on both their simulation time-step (from the shortest relaxation time), and the total length of simulation required (from the longest relaxation time). More detail on these subroutines can be found in the S1 File.

### FFEA tools

**Fig 3:**
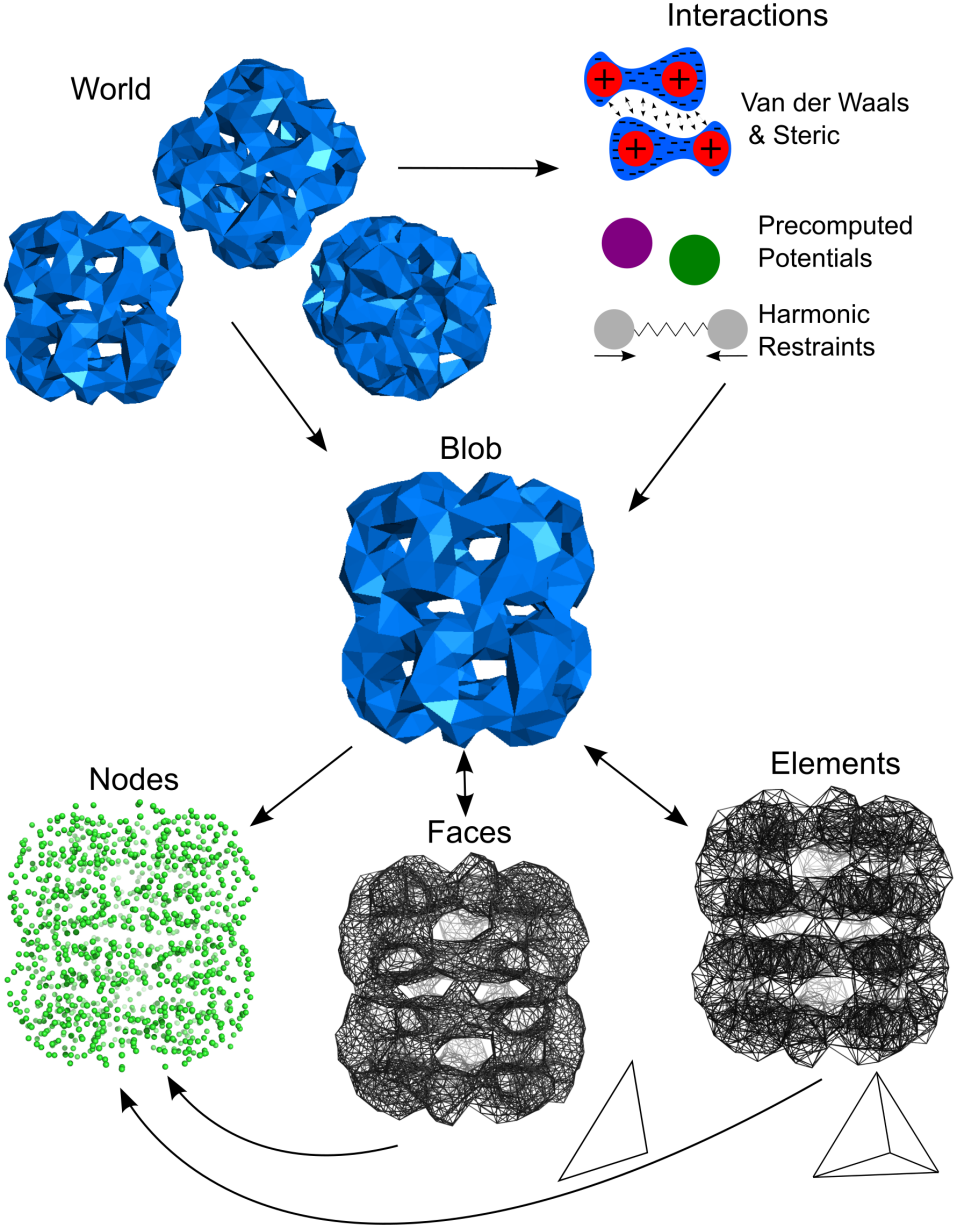
Diagram of the structure of data in FFEA. The same structure is used both in the “runner” and in the “tools”.

Accompanying the FFEA runner, we have also developed the complementary Python package ffeatools, to aid users in setting up a system for an FFEA simulation and to analyse the resulting trajectories. The core of this toolkit is a collection of Python modules that define classes associated with the various FFEA structures and file formats (Fig 3 depicts how data is structured for both the runner and the tools). These modules form the core of the toolkit, standardising the method of interfacing with the ffeatools package.

Additionally, the ffeatools package also forms the core of a set of programs centralised under a top-level script with the same name. The aim of these programs is to assist the user by automating routine operations. For example:

$$\begin{array}{c} \text{ffeatools}\;\text{pdbtoemmap}\;\text{example.pdb}\;\text{example.map}\;\text{50}\;\text{50}\;\text{50}\;\text{15} \end{array}$$

will ask ffeatools to run pdbtoemmap, a program that will take the atomic data in “example.pdb” and convert it into a 50×50×50 voxel electron density map “example.map” using an effective atomic radius of 15 Å. Each of the specific actions available from ffeatools, and their purpose, are listed in Table 1.

**Table 1:** List of actions available to the ffeatools script.

ffeatools action	brief description
pdbtoemmap	Calculate an pseudo-electron density map from atomic coordinates in PDB format
emmaptosurf	Extract a surface profile (.obj or .stl) file from an electron density map
surftocgsurf	Coarsen a surface profile whilst preserving the enclosed volume
tettonet	Convert TETGEN mesh files into NETGEN .vol files to be processed by FFEA
voltoffea	Process the volume mesh file and input material parameters into FFEA input files
split	Split a trajectory into a number of parts
thin	Select every *n* snapshots out of a trajectory
nodesFromTraj	Creates a set of .node (equilibrium) files from a trajectory snapshot
makestructuremap	Map elements into atoms (.map file)
maptraj	Calculate a pseudo atomistic trajectory given a .map file and an FFEA trajectory
pyPCAbuild	Convert the FFEA trajectory into a PDB format, allowing it to interface with the pyPcazip package
pyPCAeigen	Extract the PCA eigensystem of an FFEA trajectory
pyPCAanim	Build animations of the PCA eigenvectors
pyPCAproj	Calculate a PCA projection trace for a given eigenmode

Finally, the ffeatools modules also power the PyMOL plugin (see next section) which provides a means of visualisation for FFEA systems and trajectories. The dependency on ffeatools for all of our integrated initialisation and analysis protocols creates a self-consistent programming environment, and is essential for the maintenance of this part of the software package.

The final four tools in Table 1 allow FFEA to interface with pyPcazip [43], an open source tool designed for performing principal component analysis (PCA) on atomistic trajectories.

### Visualisation of FFEA simulations

**Fig 4:**
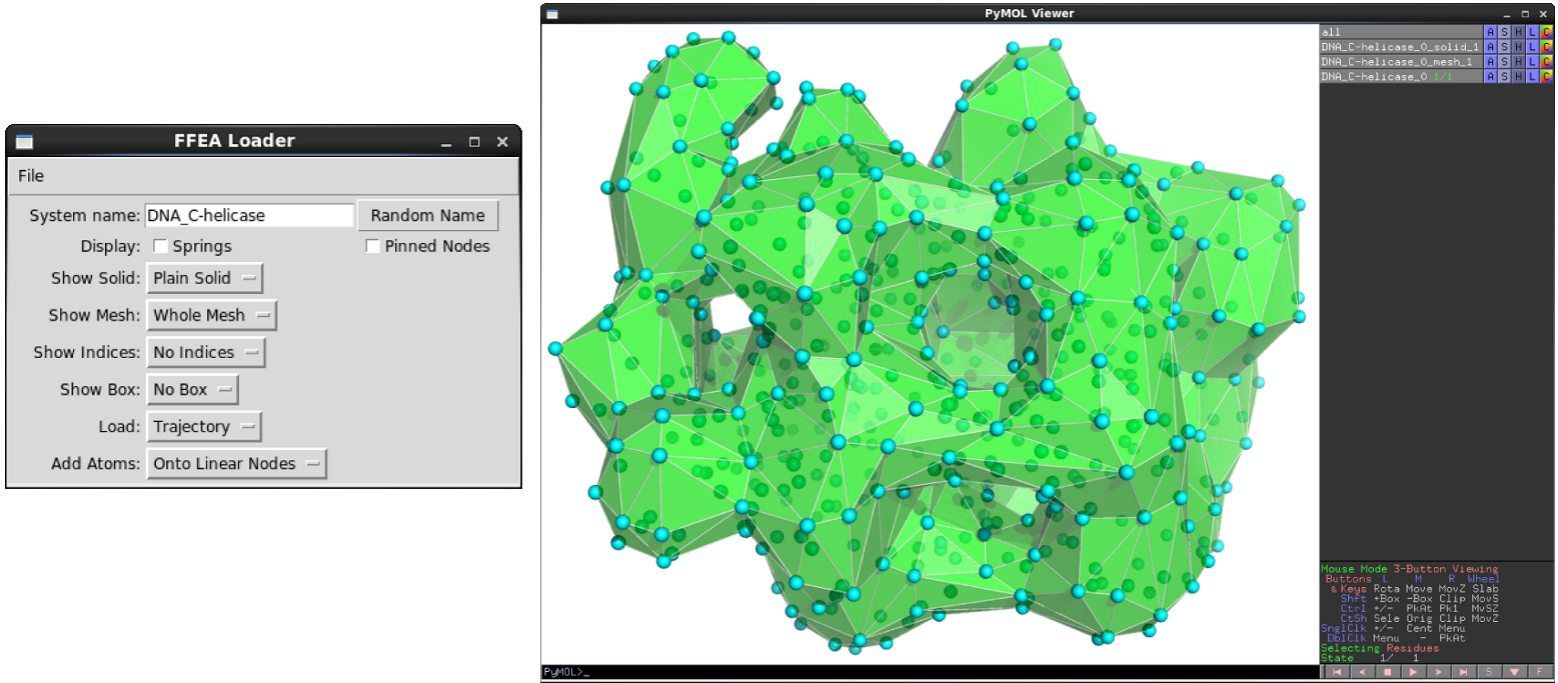
The FFEA plugin for PyMOL is displayed on the left ready to load a DNA helicase (EMDB entry EMD-2321) with “solid” faces, CGO ‘atom’ objects onto the nodes, and the 3D tetrahedral mesh, together with the FFEA trajectory named in the FFEA input file, if it is found.

We have developed a plugin for the PyMOL visualisation tool [44], which enables users to visualise both their system setup and the resulting FFEA trajectories. Prior to loading an FFEA mesh, the user can select whether to display a volumetric or surface mesh, whether to colour different bodies, according to their material parameters (Young’s modulus, shear viscosity, etc) or according to the type of surface faces (see Fig 4). Internally, these functions make use of Compiled Graphics Objects (CGO), which is the format used by PyMOL to create three-dimensional geometries. The CGO API, together with our ffeatools API, is designed to facilitate the development of further visualisation tools by multiple user groups. Within the viewer, macromolecules can be rotated, hidden, and the colours changed. Configuring springs, pinned nodes, or assigning face-face interactions requires the user to define the internal indices of the relevant nodes. While sub-structures such as nodes or faces are currently not directly selectable, a PyMOL object consisting of a set of atoms mapped onto every node, element or face, can be generated so that the residue number reflects the internal FFEA numbering. The user can then select these atoms with the mouse, and print the residue numbers to the screen.

### Atomic structures from FFEA simulations

We have also developed a software tool, atomicMapper, for the user to convert from the continuum FFEA trajectory to an estimated trajectory for the corresponding atomistic structure, provided that atomic structural information is available. Geometrically, a biomolecule within FFEA is simply a set of nodes connected topologically as a set of tessellating tetrahedra. During the simulation the coordinates of the nodes are continuously changing, while its topology remains constant. This enables us to relate the positions of the atoms comprising the biomolecule to the coordinates of the nodes via a linear interpolation scheme.

## Results

The FFEA package contains detailed documentation on the software, including installation instructions, in-depth descriptions of the capabilities of the software, a documented API, and a tutorial on how to set up a simulation. We also provide a test suite to validate our software implementation and installation. These tests examine both the computational and physical accuracy of FFEA following installation, and should be run to ensure that a local installation of the FFEA runner is functioning correctly.

To demonstrate the capabilities of FFEA, in our tutorial we demonstrate the full initialisation and simulation procedure for the molecular chaperone GroEL in its “apo” state, a macro-molecular structure for which a fully-atomistic simulation would be extremely computationally demanding. Analysis of the output data showed that the resulting dynamical information is physically meaningful and can aid in the understanding of macromolecule function.

We have also performed a comparison between all-atom MD simulation trajectories and equivalent FFEA simulations of two different proteins. Through this analysis, we aim to show how much dynamical information is preserved as we remodel the system in the FFEA framework.

### A simulation of apo-GroEL

The tutorial uses the molecular chaperone GroEL as an illustrative example to guide the user through setting up and performing an FFEA simulation. GroEL consists of two heptameric rings built of 14 identical subunits, in total weighing approximately 770kDa. With this weight, and at 12 × 14 × 14 nm in size, GroEL would certainly be considered large for conventional atomistic MD simulations with standard computational resources. Structural data is available both at atomic resolution (PDB entry 4HEL), and at lower resolution as an electron density map (EDM) (EMDB entry EMD-5403).

**Fig 5:**
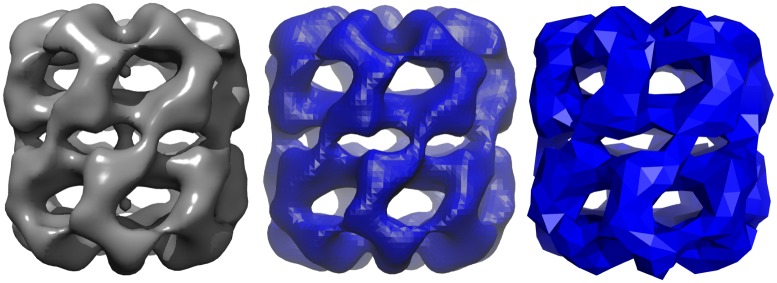
An electron microscopy map of GroEL (EMDB entry EMD-5403) as seen in Chimera [26](left), then converted to “.stl” using the emmaptosurf ffeatool visualised in VMD [27] (centre), and the final volumetric mesh, coarsened so that the shortest edge is 12Å visualised using the FFEA plugin for PyMOL (right).

One of the strengths of FFEA is that it is able to to build a continuum structure from low resolution data. To illustrate this, we began our initialisation procedure from the EDM (see Fig 5, left) and extracted a wavefront “.obj” file, a connected triangulated surface file, using ffeatools:emmaptosurf (see Fig 5, centre). This was then coarsened using ffeatools:surftocgsurf (which implements an algorithm described by Oliver [45]) until the shortest edge in the system was was 12Å, maintaining constant volume. The resulting STereoLithography triangle mesh “.stl”, a standard file format for defining a triangulated surface, is then read into the Tetgen software package to create a volumetric mesh, completely filling the structure with tessellating tetrahedra (Fig 5, right). We emphasise that to create this volumetric mesh, Tetgen (or alternatively Netgen) requires that the provided surface is *closed* i.e. a surface with a well defined and completely separate ‘inside’ and ‘outside’.

For demonstrative purposes we have performed two separate simulations of GroEL, which will be labelled using the index *i*. Each simulation had a different value of the Young’s modulus, *E*_*i*_, which were *E*_1_ = 0.33GPa and *E*_2_ = 1GPa. The complementary parameter, the Poisson ratio, was kept constant at *ν* = 0.35 for all simulations. These values are representative of generic globular proteins [46]. In addition to these core parameters, we assigned the internal and external viscosity values, *μ* = 10^−3^ Pa⋅s, equivalent to that of water. More generally, material parameters can either be inferred from experimental results, such as flexibility information derived from negative stain EM images, or calculated though atomic simulations of constitutive protein fragments. The simulations were performed using the Brownian formulation, i.e. using Eq 3 as the equation of motion, which allowed us to use a simulation time-step, *dt* = 0.1*ps*. We performed 3*μ*s FFEA simulations and recorded the shape of the FE mesh representing the protein complexes every 1ns for post-processing. These simulations took approximately 65 hours using 4 cores of an AMD 6376 Opteron processor at 1.4GHz.

### Analysis of apo-GroEL

**Fig 6:**
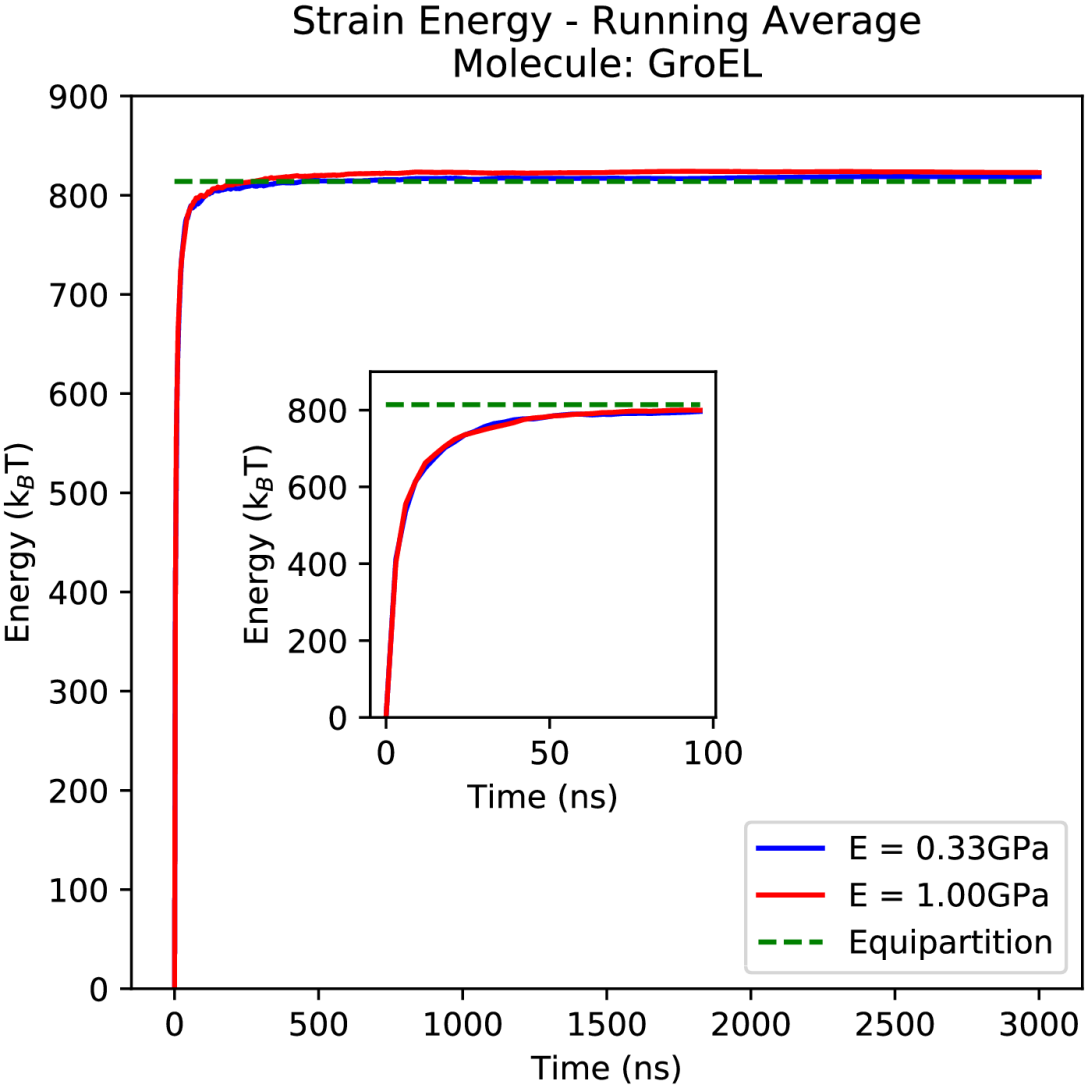
The average strain energy of the apo-GroEL simulations plotted as a function of the length of time the average was taken over. The inset graph shows the first 100ns at higher resolution.

To validate the simulations of GroEL, we measured the average strain energy over the course of the simulation, 〈*U*(*t*)〉, where *t* is the length of time used for the averaging. Fig 6 shows that the strain energies successfully converged within approximately 50*ns* (inset) and remained equilibrated for the remainder of the simulation. Simulations 1 and 2 converged to within 0.60% and 1.09% respectively of the theoretical value $\langle U\rangle =\frac{1}{2}k_{B}T$ per degree of freedom as predicted by the equipartition theorem, where *k*_*B*_ is Boltzmann’s constant. We see a small increase in the numerical inaccuracy with larger Young’s moduli due to the corresponding decrease in the system relaxation times.

**Fig 7:**
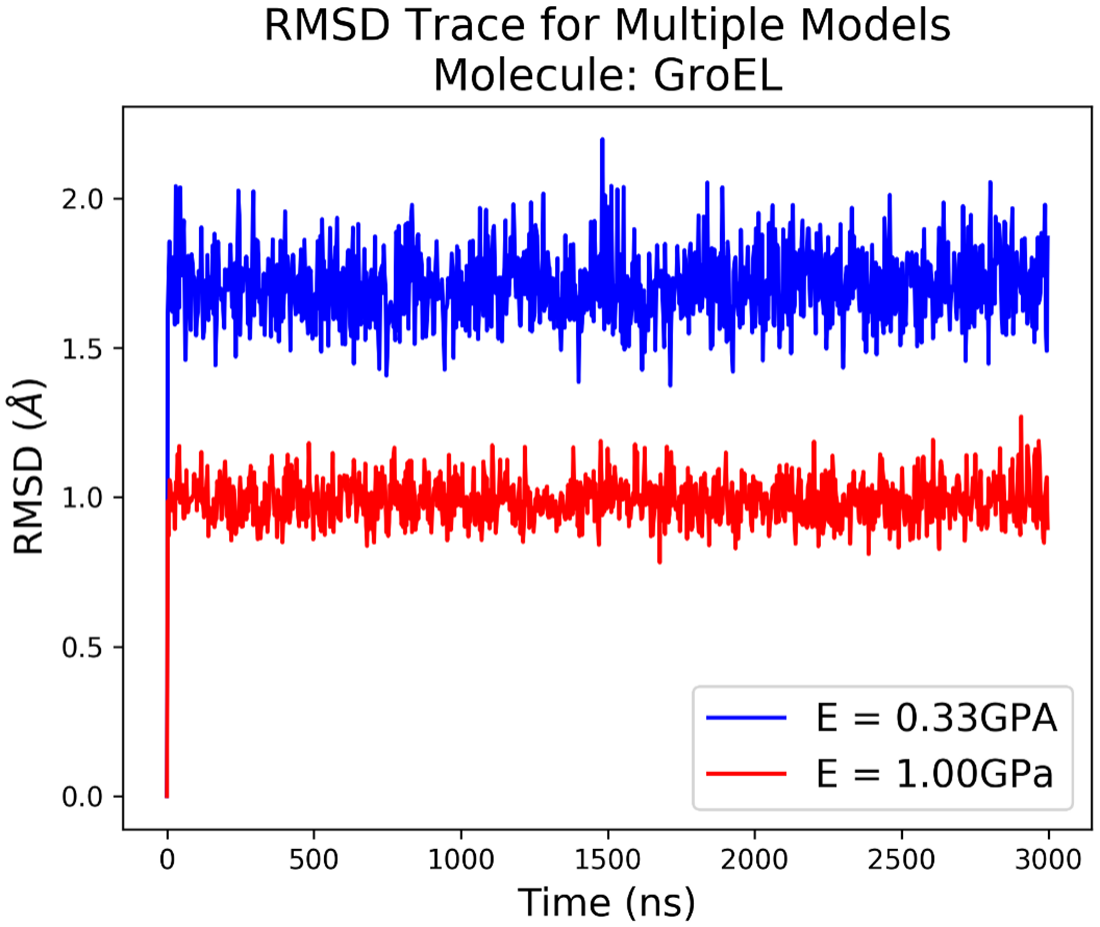
The RMSD traces of the 2 different simulations of GroEL following an RMS fit to the average structure.

By calculating the Root Mean-Squared Deviation (RMSD) throughout the simulation using the nodes of the finite element mesh, we can see the difference in dynamics due to the differing elasticities. Fig 7 shows these RMSD traces following an RMS fit of each frame in the trajectory to the average structure. We see that as we increase the stiffness both the size of the fluctuations in the nodal RMSD, as well as the average value, both decrease, showing that the molecule is more tightly restricted to its average structure.

**Fig 8:**
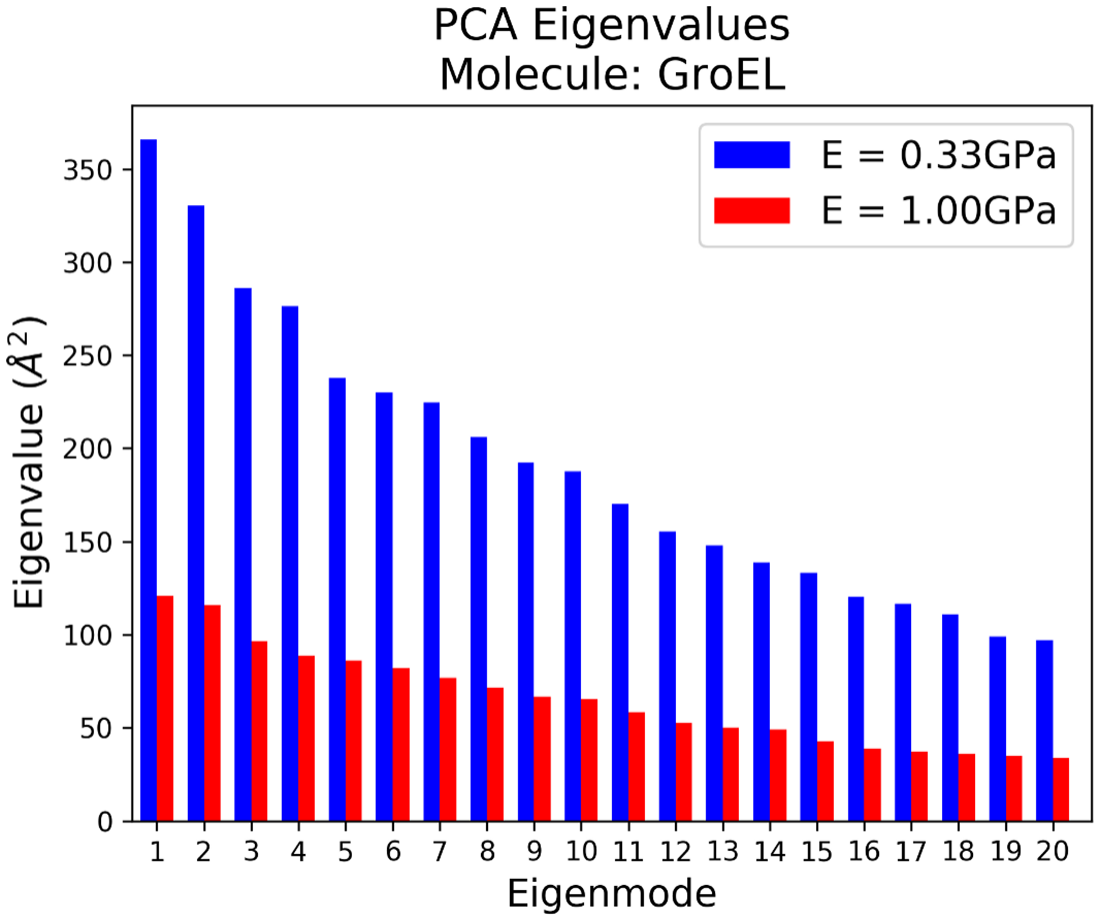
The eigenvalues, which correspond to positional variance, of the 20 most flexible modes found from PCA analysis of a 3*μ*s FFEA simulations of GroEL.

To further probe the dynamics of GroEL, we performed PCA on the trajectories to determine the major types of motion. Using the pyPcazip interface (see Section: FFEA tools), we were able to determine the 20 most flexible normal modes of motion from the FFEA trajectory, the eigenvalues of which are shown in Fig 8. The factor 3 difference in the Young’s moduli between simulations 1 and 2 gives the same factor 3 difference between the eigenvalues, which represent the flexibility of the molecule. We also note that in each individual simulation, many of the eigenvalues are similar in magnitude, indicating a near degeneracy of several of the eigenmodes. This is to be expected given the substantial symmetry of the GroEL structure. All the relevant trajectories and analysis files were made available within S1 Text.

**Fig 9:**
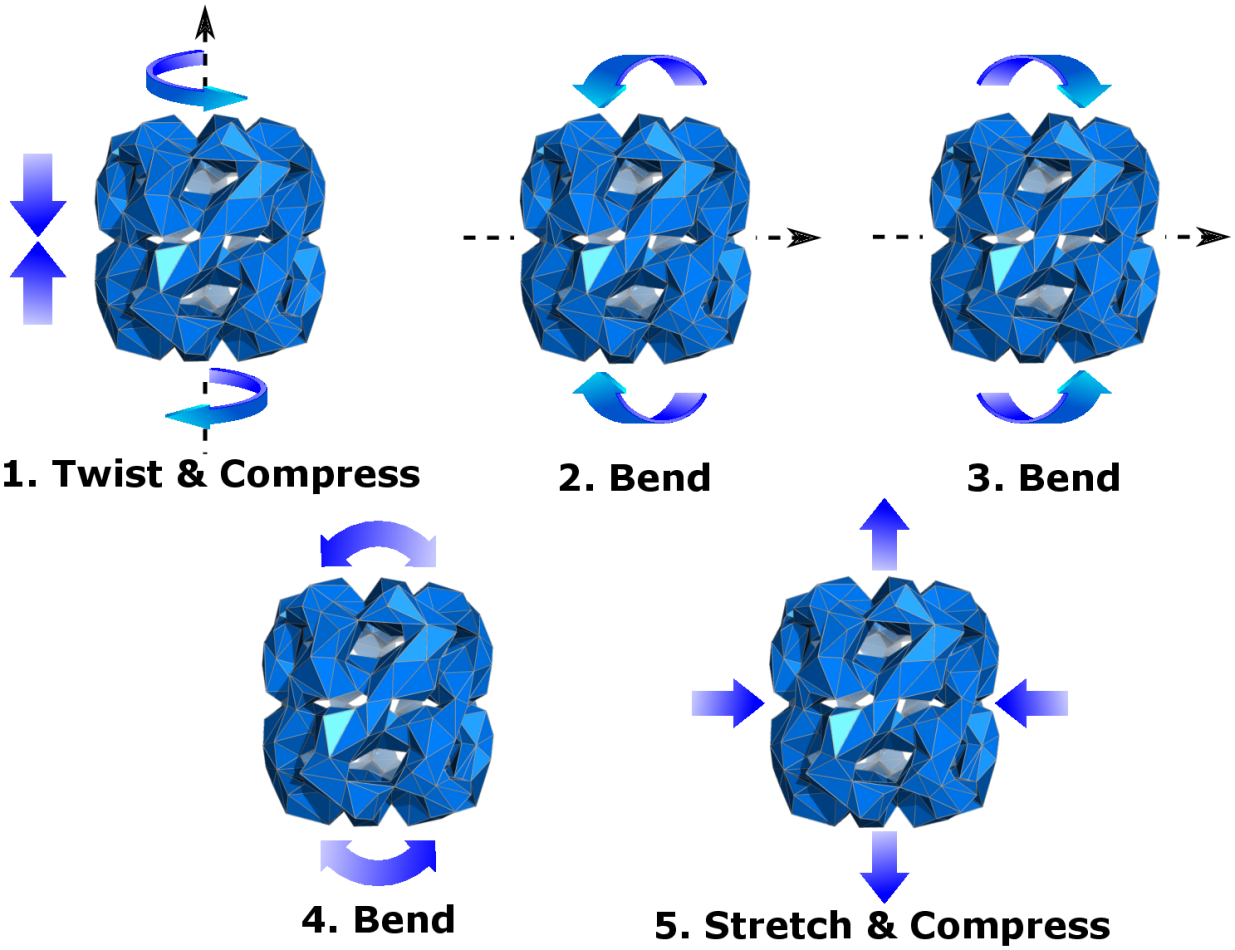
A representation of the 5 most flexible eigenmodes of the GroEL 1GPa model.

Our interface with pyPcazip also provides functionality for visualising these modes in FFEA format. The 5 most flexible modes are shown in Fig 9, some of which exhibit degeneracy as mentioned above.

### Recovering atomic structures

**Fig 10:**
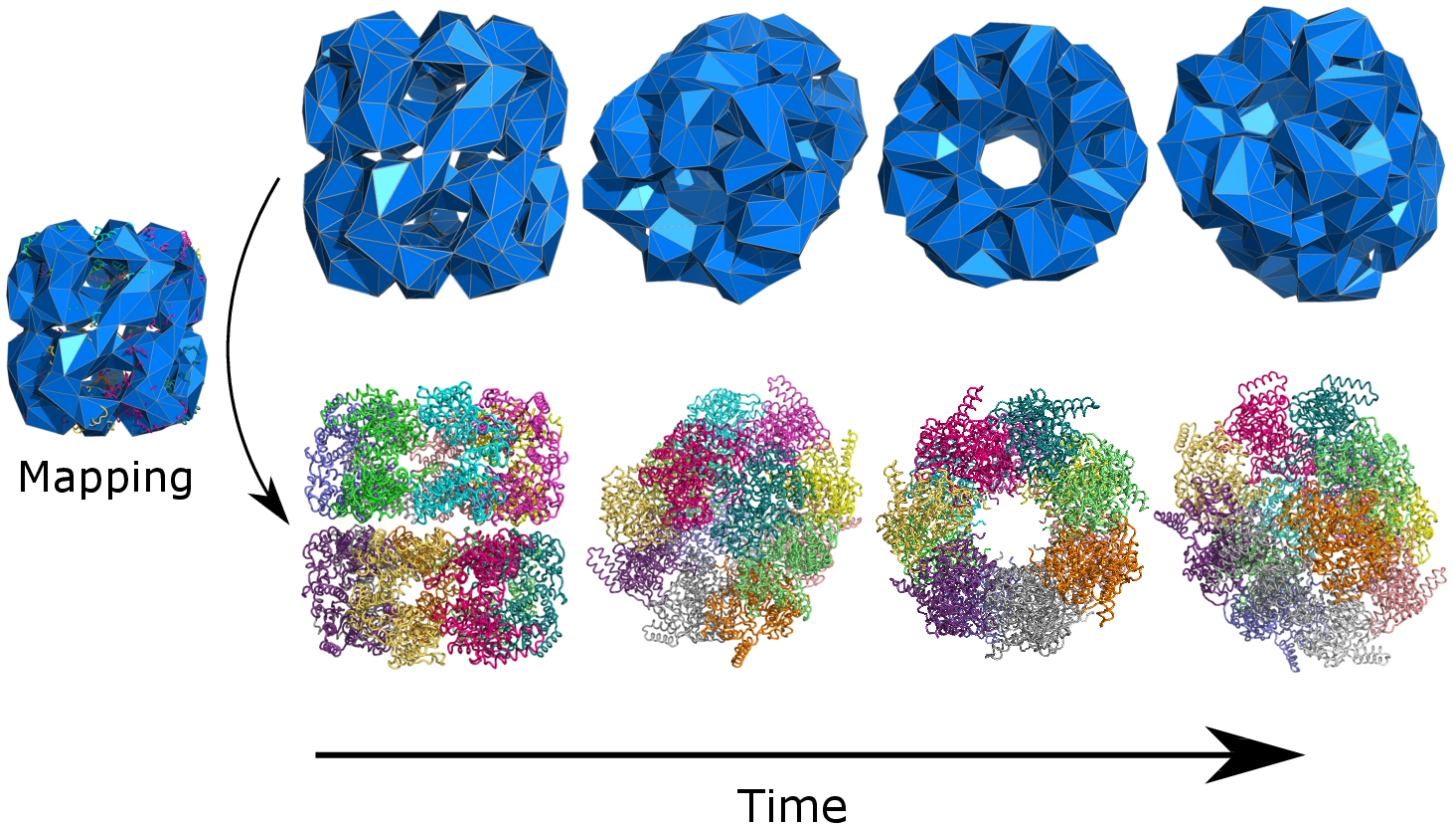
Four snapshots from the FFEA trajectory of GroEL and the atomistic pseudo-trajectory formed from the mapping procedure.

**Fig 11:**
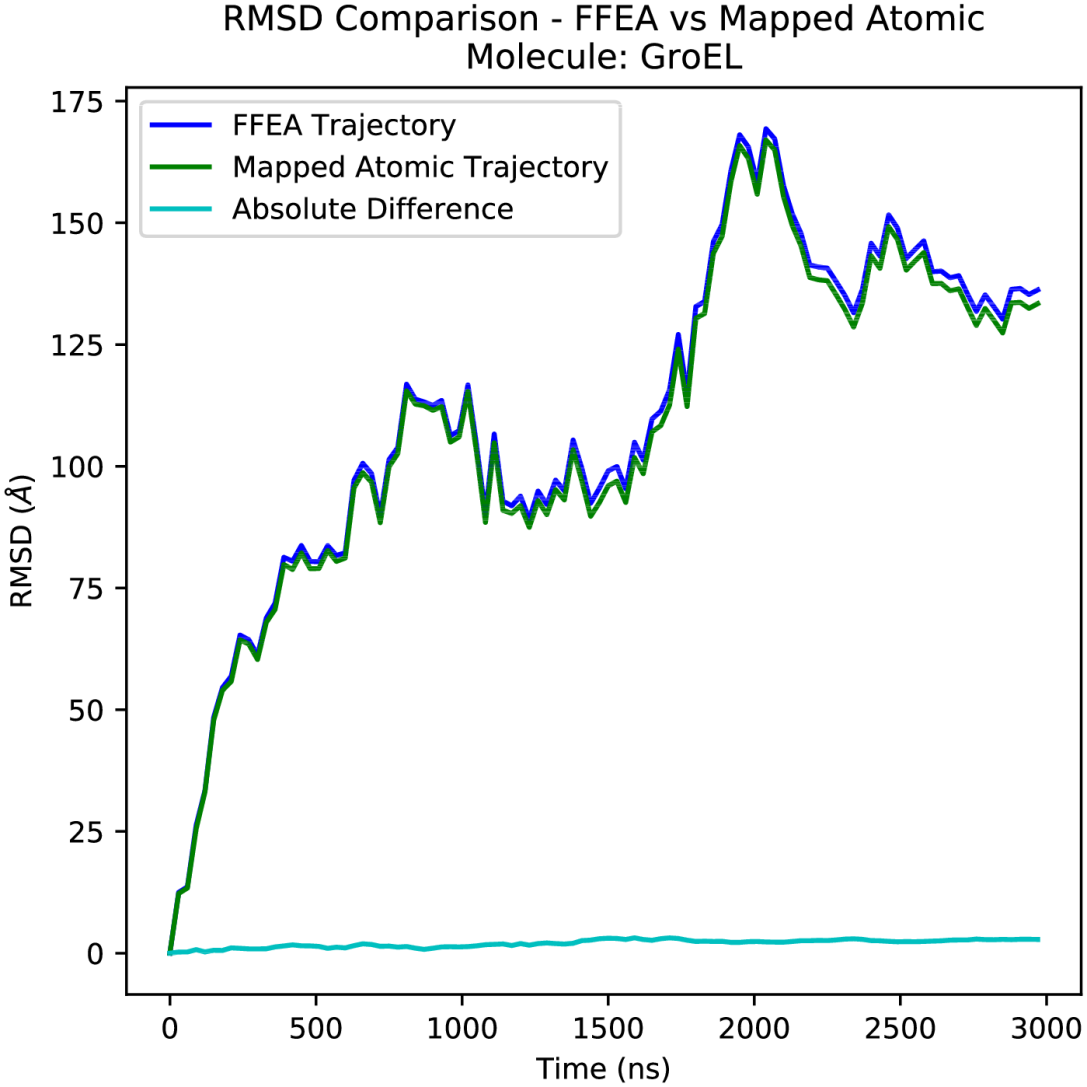
Root Mean-Squared Deviation traces of an FFEA simulation of GroEL (calculated using the *nodes*), and the pseudo-atomic trajectory created via the atomistic mapping procedure.

In order to demonstrate the functionality of the atomicMapper tool, we performed the mapping procedure on the *E* = 1GPa GroEL trajectory to produce an equivalent atomistic trajectory that conserves the FFEA calculated dynamics, visualised in Fig 10. The time evolution of the RMSD of both the FFEA trajectory and the mapped atomistic counterpart, calculated using the *nodes* and atoms respectively, can be seen in Fig 11. No alignment has been performed on the frames with respect to the initial structure, meaning that these RMSDs still contain the diffusional dynamics of the system within them. We see negligible difference between both trajectories, showing that physically meaningful measurements are conserved through the mapping procedure.

**Fig 12:**
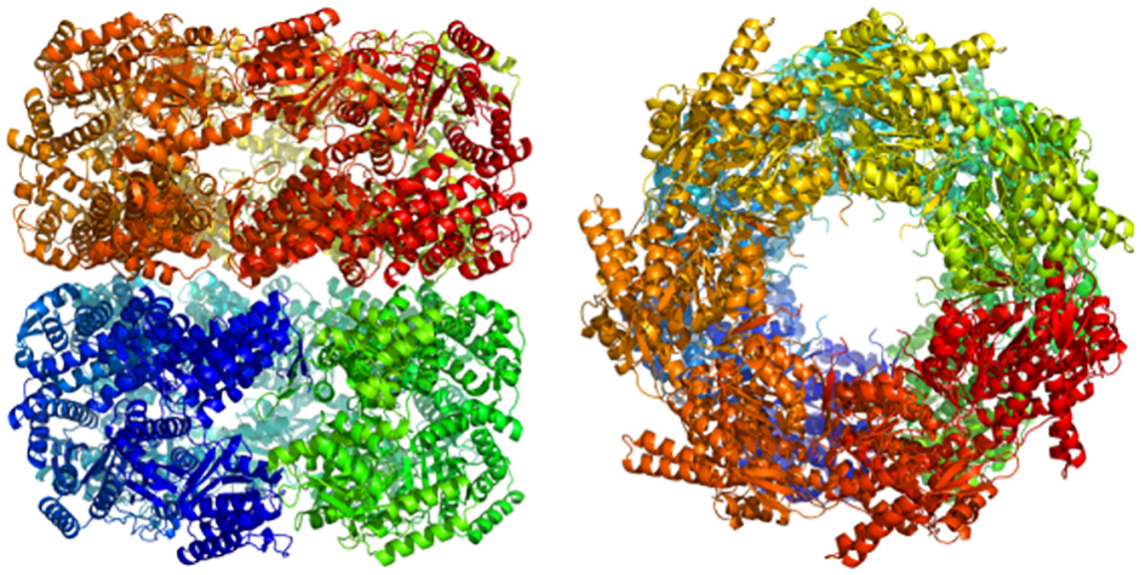
The minimised structure of fully atomistic GroEL following the mapping procedure.

The mapped atomic structures at each time-step were then suitable for higher resolution, atomistic modelling. In Fig 12 we see an atomic structure which was created using the atomicMapper following 1*μ*s of FFEA simulation, and minimised using the AMBER MD package. This multi-resolution scheme could potentially be used to rapidly explore conformational space using a continuum FFEA model, before transforming back to an atomic resolution simulation to explore the dynamics of the new configuration in more detail.

### Comparison of FFEA with all-atom molecular dynamics

**Fig 13:**
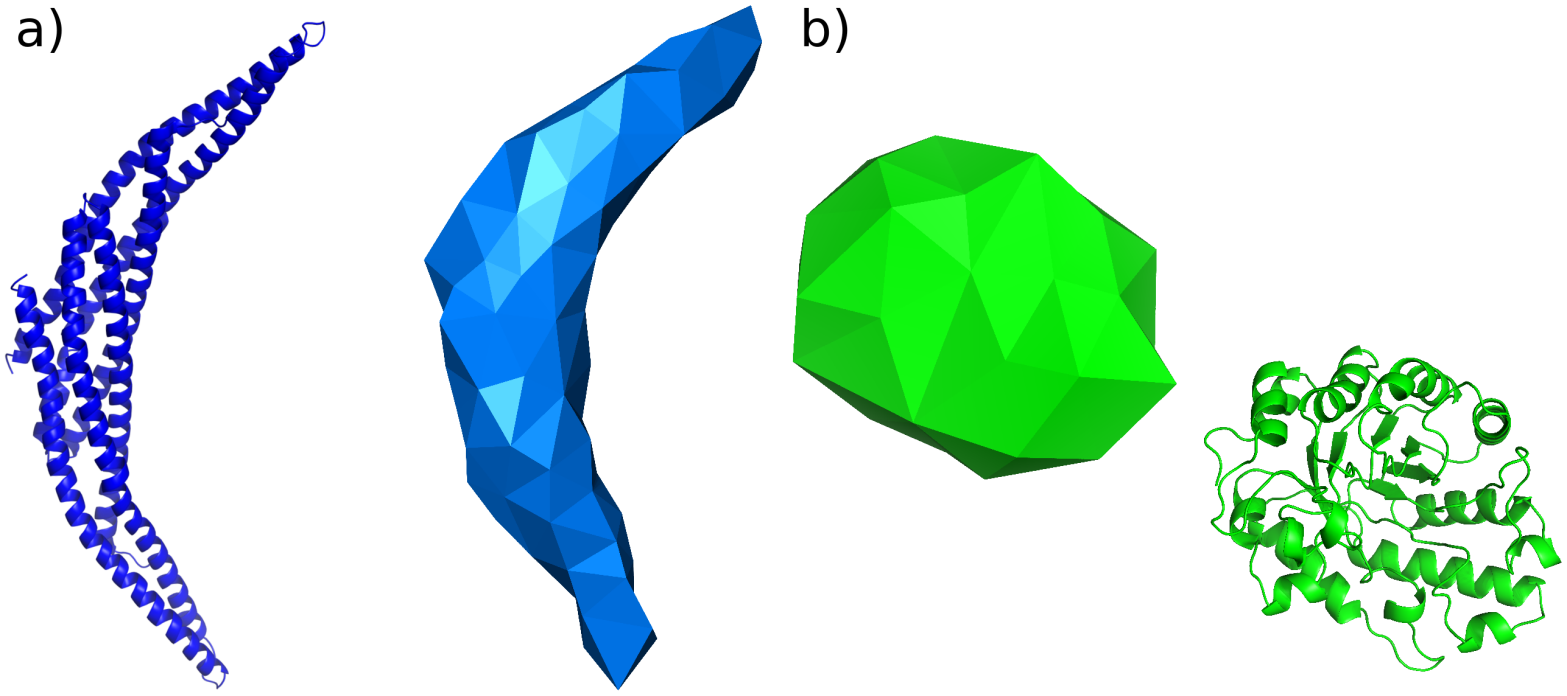
The two molecules used for our FFEA / atomistic comparative study: a) Arfaptin, and b) xylanase.

From the MoDEL database [47], we obtained PCA compressed atomistic trajectories for two small proteins (Arfaptin, PDB ID 1I49 [48], and xylanase, PDB ID 1TUX [49]). These proteins were chosen due to the contrasting dynamical behaviour observed in the first principal components extracted by PCA. For Arfaptin, which is long and thin, the first principal component extracted from the atomistic simulations involved a motion that is delocalised over the entire protein, whereas for the spherical protein xylanase, the first principal component was associated with local motions of individual side chains. Consequently, we expect that FFEA is able to capture the dynamics observed in atomistic simulations of Arfaptin, but not of xylanase. The atomistic and FFEA structures are shown in Fig 13, the animations of selected principal modes are available as Supplementary Information in S2 File, as well as the trajectories and analysis files within S2 Text.

**Fig 14:**
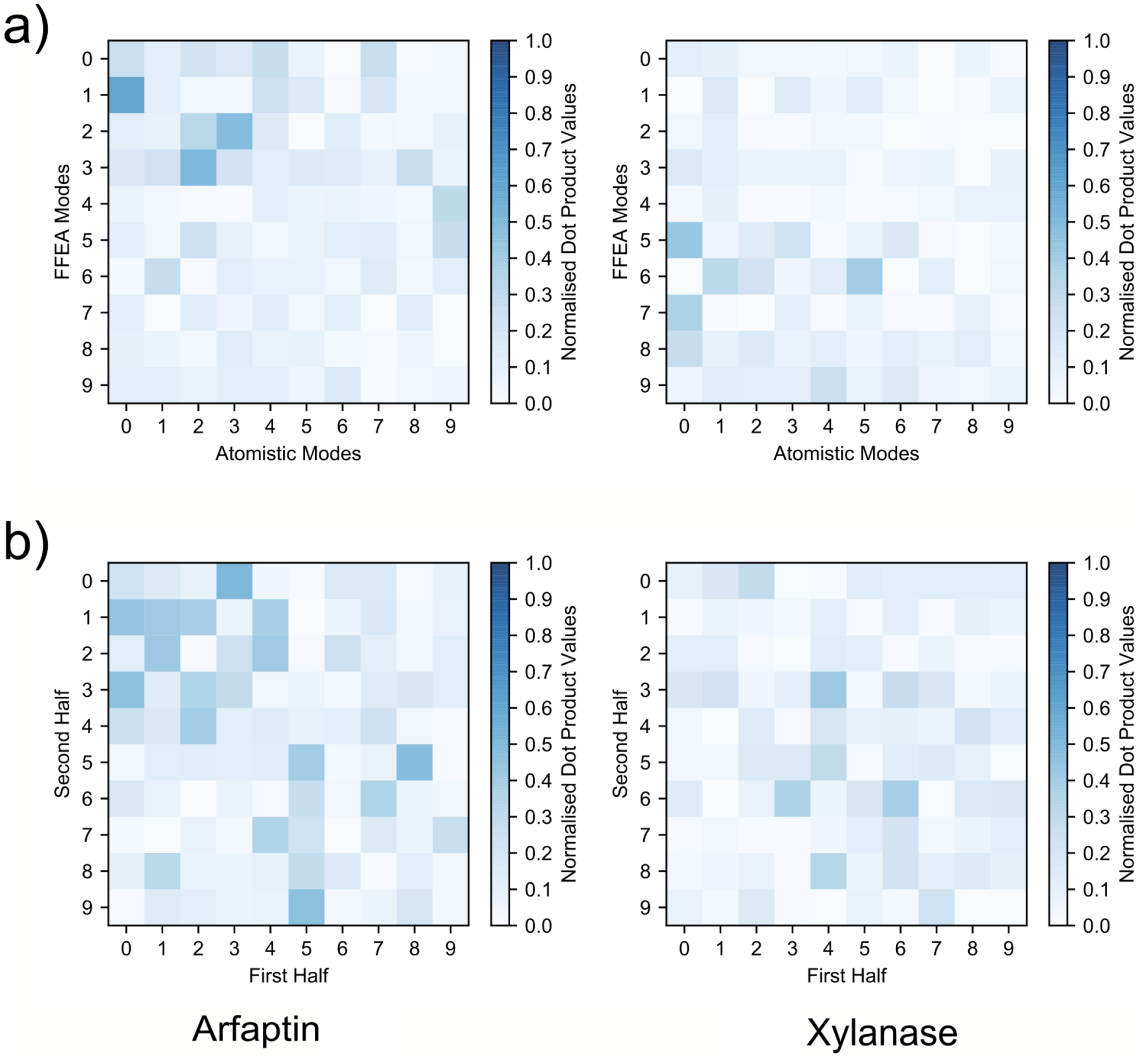
The eigenvector inner product matrices between compared trajectories. **a)** is the comparison between atomistic and FFEA pseudo-atomistic PCA datasets. **b)** is the comparison between the first and second halves of the atomistic datasets.

In the FFEA simulations, we assigned homogeneous material parameters of *E* = 1GPa for the Young’s modulus, a Poisson ratio 0.35 and shear, bulk and external solvent viscosities *μ* = 1 × 10^−3^Pa.s. The FFEA simulations were run for 100ns for Arfaptin and 780ns for xylanase, which corresponds to an order of magnitude longer than the atomistic trajectories in each case. Using the mapping procedure described previously, we transformed each of the resulting FFEA trajectories into pseudo-atomistic trajectories before performing PCA with the pyPcazip software [43]. Performing this transformation from the FFEA model onto the atomistic structure allows us to directly compare the dynamics of the two types of simulation trajectory on a common coordinate system. For each model, we compared the atomistic and FFEA pseudo-atomistic PCA eigensystems by calculating the inner product matrices of the most flexible 10 eigenmodes from each data set using the pyPczcomp utility of pyPcazip. These matrices are shown in Fig 14a. As expected, while the atomistic and FFEA simulations exhibit common dynamical modes for the global modes of Arfaptin (eigenspace overlap of 0.6), for the xylanase trajectories, which involve only local structural changes, the continuum and atomistic representations show less correlation (eigenspace overlap of 0.4). We also performed an eigenvector comparison between the first and second halves of the atomistic trajectories, for which the inner product matrices are shown in Fig 14b (eigenspace overlaps 0.7 for Arfaptin, and 0.5 for xylanase). A comparison of the first and second halves of the atomistic simulations for Arfaptin shows that the FFEA model provides a similar level of agreement to different segments of an atomistic simulation.

### FFEA code performance

We benchmarked FFEA on two different machines by performing simulations containing an increasing number of GroEL units. The first machine is a single compute node, with two Intel Xeon E5-2640v3 2.60GHz processors, providing a total of 32 hyperthreaded (HT) cores, with 32Gb of memory (DDR4, 1866MHz). Using a single core, FFEA provides 18 ns/hour for a single GroEL, which is comprised of 3×3109 degrees of freedom. The best performance was achieved using all 32 available cores, which ran at a speed of 129.6 ns/hour.

**Fig 15:**
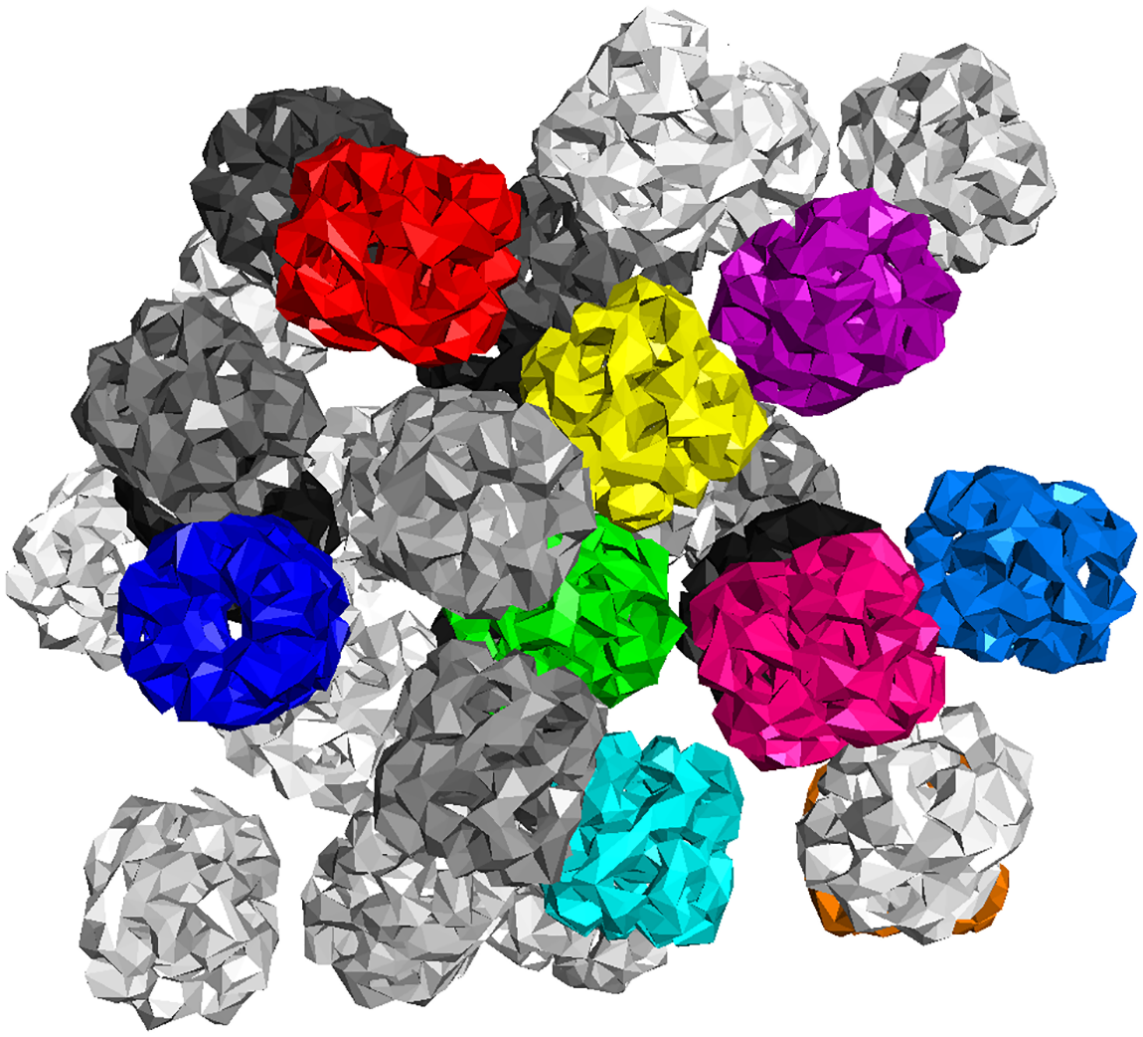
Arrangment of 32 GroEL units used to benchmark the performance of the FFEA runner.

**Fig 16:**
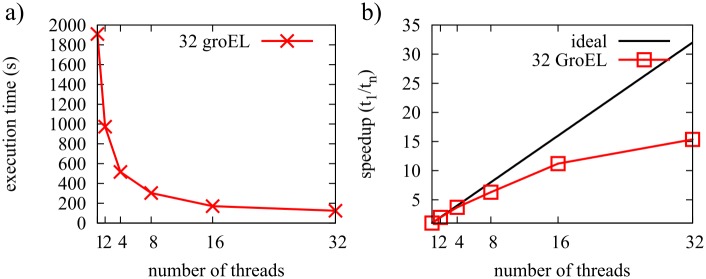
Strong scaling plots for a system of 32 GroEL complexes, run using a 32 HT core computer. a) Execution time b) Speedup.

We then benchmarked FFEA for a simulation cell composed of 32 GroELs (as shown in Fig 15). This task is more computationally challenging, because steric interactions between faces belonging to different proteins need to be included to prevent different molecules occupying the same space within the simulation box. Fig 16 shows that while the performance increases up to 32 cores, the level of improvement diminishes for higher core numbers.

**Fig 17:**
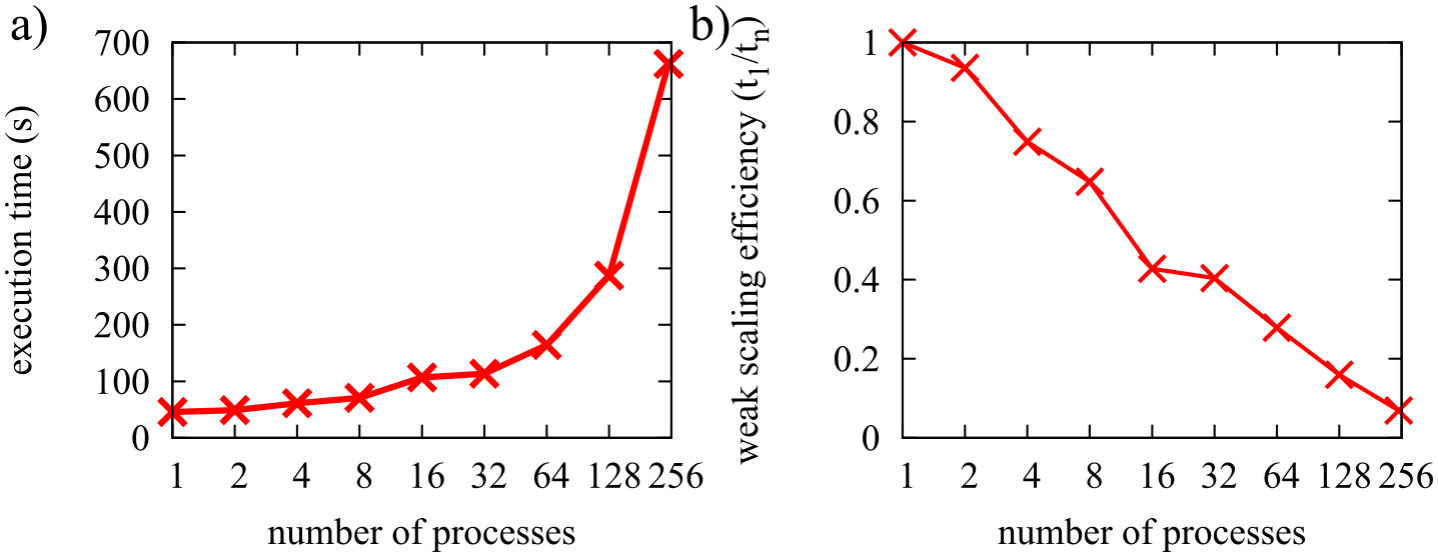
Weak scaling plots for a system composed of an increasing number of GroEL units matching the number of processors used, up to 248, in a large shared memory machine. a) Execution time b) Efficiency.

The second machine we used for benchmarking FFEA is an SGI UV2000 shared memory computer, with 32 nodes composed of an Intel E5-4650L processor (8 cores, 2.6GHz) and 8 DIMMs of 16Gb of memory (1600MHz, DDR3) each, resulting in a total of 256 cores and 4Tb of memory. One node is reserved as the login blade, so we used up to 248 cores. To study the weak scaling performance of FFEA, we loaded the machine with increasing numbers of GroEL proteins so that one GroEL could be assigned to each thread. The results in Fig 17b show how the efficiency is reduced when increasing numbers of proteins are placed on successively more cores. The performance reduction is a result of the larger load per thread, due to the increased number of face-face interactions that need to be considered. While the weak scaling benchmarks show that the efficiency is reduced to 7% when using 248 cores in 31 blades, we are still able to simulate as many as 248 GroELs. Consequently, it is possible, if expensive, to simulate a very large biological system with FFEA.

### FFEA validation

The FFEA package is released with a test suite that should be run by the user following successful compilation. Tests can be run either sequentially (by typing “make test”) or concurrently (by typing “ctest -j N”, where *N* is the number of processes) reporting either failure or success.

This test suite, summarised in Table 2, provides structural checks of the FFEA software itself as well as validation of the physical results calculated by the code.

**Table 2:** List of tests included in the FFEA tests suite.

test name	brief description
test_rngStream	unit tests on the random number generator
test_volume	the intersection volume of two tetrahedra is checked against a known geometry
test_script_loader	test of the input file reader for the “runner”.
python_load_trajectory	test of loading a trajectory using the ffeatools.
restart_check	restart check proving that the RNG used to simulate the thermal noise is used safely
precomp_check	calculate the energy for a number of beads interacting through pre-computed potentials and compare it with the value obtained using Gromacs [36].
sphere_mass†	showing correct distribution of thermal energy for the Langevin scheme
sphere_nomass†	showing correct distribution of thermal energy for the Brownian scheme
sphere_diffusion_mass†	showing correct diffusional properties for the Langevin scheme
sphere_diffusion_nomass†	showing correct diffusional properties for the Brownian scheme
cyl_youngs_mod†	mechanical stretching of a cylindrical beam
cyl_flexrig†	mechanical bending of a cylindrical beam
steric_n_springs†	test of springs and steric interactions

^†^Tests explained in detail in the main text.

#### Tests of thermodynamic properties

In order to check that the systems equilibrate to the correct energy distributions and thus respond correctly to the thermal fluctuations, short trajectories of single spheres are run under the Langevin and Brownian schemes. These spheres have radius *R* = 5nm, a Young’s modulus *E* = 1GPa, Poisson ratio *ν* = 0.35 and viscosity *μ* = 10^−3^ Pa⋅s. The trajectories are then analysed and the average energy of the last 60 recorded steps is calculated for the different terms. These are strain energy and, for the Langevin scheme, kinetic energy, and their averages are compared to the theoretical value given by the equipartition theorem (*k*_*B*_ *T*(3*N* − 6)/2 for the strain energy, and 3*k*_*B*_ *TN*/2 for the kinetic energy). A test of 10^5^ time-steps (simulating 1ns in less than 20s of CPU time) is sufficient to give an accuracy of 0.6% for the strain energy in the Brownian scheme, and 1.2% for the strain energy and 2.9% for the kinetic energy using the Langevin scheme. Averages using 20ns trajectories give better results, lowering to 0.07% for the Brownian strain, and to 0.03% for the strain energy and 0.1% for the kinetic energy in the Langevin case. We suspect that the kinetic energy does not converge more accurately to the theoretical value due to the time-stepping algorithm used here, in agreement with previous studies (see [50] for a comprehensive review). Future work on improving the algorithm for the integration of the stochastic differential equations, especially in the Langevin scheme, is planned in order to provide greater accuracy together with potential increases in speed. This should allow larger simulation time-steps whilst at the same time preserving the stability of the numerical integration and thermodynamic correctness of our thermal stress.

#### Tests of FFEA mechanics

Two tests are provided to validate the mechanical calculations within FFEA. These perform simulations that separately stretch and bend a cylinder, 160 nm long, with a 10 nm radius and Young’s modulus of 600 GPa. In both cases, one end of the cylinder has all its nodes pinned, and constant force is applied on the other end, spread uniformly over the end surface.

In the stretching test, a force *F* = 10 pN is applied in the same direction of the cylinder axis. When the system reaches equilibrium the increase in length of the cylinder, Δ*L*, is measured and the Young’s modulus is calculated using its definition: *E* = *FL*_0_/*A*_0_Δ*L*, where *L*_0_ and *A*_0_ are the equilibrium length and cross section area respectively. This is then compared with the Young’s modulus used to parametrise the system, resulting in error smaller than 0.2%.

In the bending test, we compare computational and theoretical results for the *flexural rigidity* of the beam, *EI*, i.e. its resistance to bending. From beam theory, a beam with a free end where a force is applied perpendicular to its axis, suffers a deflection of Δ*y* = −*FL*^3^/3*EI* [51], where *I* is the second moment of the cross-sectional area of the beam, a geometrical property. Computationally, the test runs a trajectory for the same cylinder, where a constant force of 1 pN is now applied in a direction perpendicular to the cylinder axis. When the cylinder reaches equilibrium, Δ*y* is measured, and the corresponding *EI* is compared to the theoretical values. However, in this case, errors depend on the quality of the mesh, the reason being that linear elements restrict bending motion, and therefore coarse meshes are stiff compared to the true continuum. In the mesh used and shipped in the test suite, the error is 4%, but coarser meshes will be less accurate [25].

#### Validating diffusion in FFEA

A test is provided for both the Langevin and Brownian schemes to check that the simulated diffusion of a biomolecule is correct. In each test, a 10 ns simulation is performed on a spherical object, of radius *R* = 5 nm, and the diffusive behaviour compared with the theoretical value.

The viscosity of the solvent results in a drag on the solute which we apply locally to each node. The nodes are modelled as spheres with an effective radius, *r*, embedded in a fluid of viscosity *μ*^*s*^, as explained in S1 File. Choosing $r=\frac{R}{N}$, *R* and *N* being the radius of the sphere and the number of nodes respectively, makes the total drag of the object λ = 6*πRμ*^*s*^, the expected result for a spherical object. In addition to these viscosities, each sphere has density, *ρ* = 1500kgm^−3^, Young’s modulus, *E* = 1GPa and Poisson’s ratio, *ν* = 0.35.

For the Brownian scheme, the diffusion has the well known form:

$$\begin{array}{c} \left\langle r^{2}\right\rangle =6\frac{k_{B}T}{\lambda }t \end{array}$$ (6)

For the Langevin scheme however, inertial effects need to be taken into account. For mesoscale objects like our sphere, the ballistic motion only occurs for short periods of time before the background viscosity dominates. In this regime, the approximation 〈(*x* − *x*_0_)^2^〉 = 〈*v*^2^〉*t*^2^ is valid, and using the equipartition theorem *m*〈*v*^2^〉 = *k*_*B*_ *T*,

$$\begin{array}{c} \left\langle r^{2}\right\rangle =3\frac{k_{B}T}{m}t^{2} \end{array}$$ (7)

Short simulations are performed using time-steps small enough to retain numerical accuracy (see S1 File). Following equilibration, we calculate 〈*r*^2^〉 per time-step by averaging (*r*_*t*+Δ*t*_ − *r*_*t*_)^2^ for 0 < *t* < *t*_*sim*_, where *t*_*sim*_ is the total simulation time. We report errors of 2.73% for the inertialess solver, and 8.39% for the inertial solver. These errors differ by ∼ 0.1% between tests due to the random nature of the stochastic noise over short simulations. The higher overall error value for the inertial solver is expected, and is again likely a consequence of our numerical integration scheme.

#### Tests of the steric repulsion within FFEA

**Fig 18:**
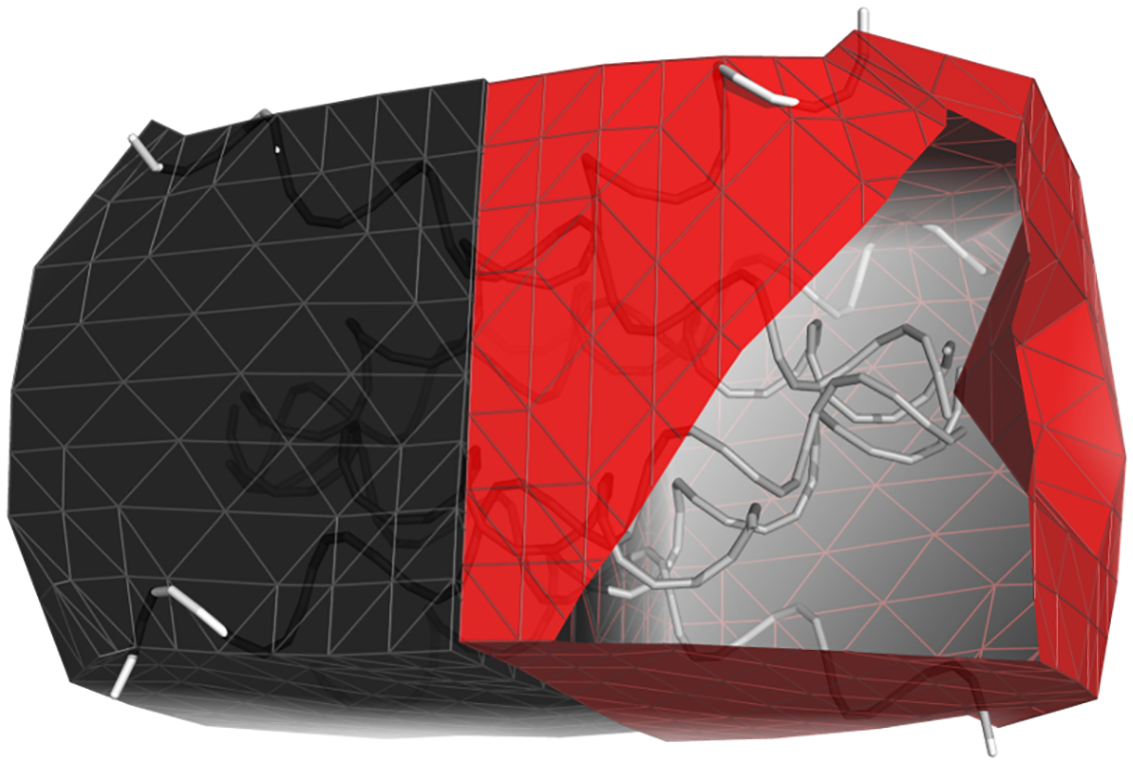
Two viscoelastic cubes are put side by side with springs pulling from both ends in the configuration displayed (where part of the red cube was made transparent) to test the effectiveness of the steric repulsion introduced. As it can be seen, the interface between both bodies remains flat, while the overall volume is compressed.

Finally, simulations of viscoelastic cubes in the absence of thermal noise are performed to ensure that the steric repulsion is correctly implemented in both the Langevin and Brownian schemes. For the Brownian scheme, two viscoelastic cubes are placed in face-to-face contact and are pulled together by springs (see Fig 18) during the simulation to test the intermolecular forces. The resulting trajectory is analysed automatically, checking that the centers of mass of both cubes approach progressively while the overlapping volume between the meshes remains approximately zero.

**Fig 19:**
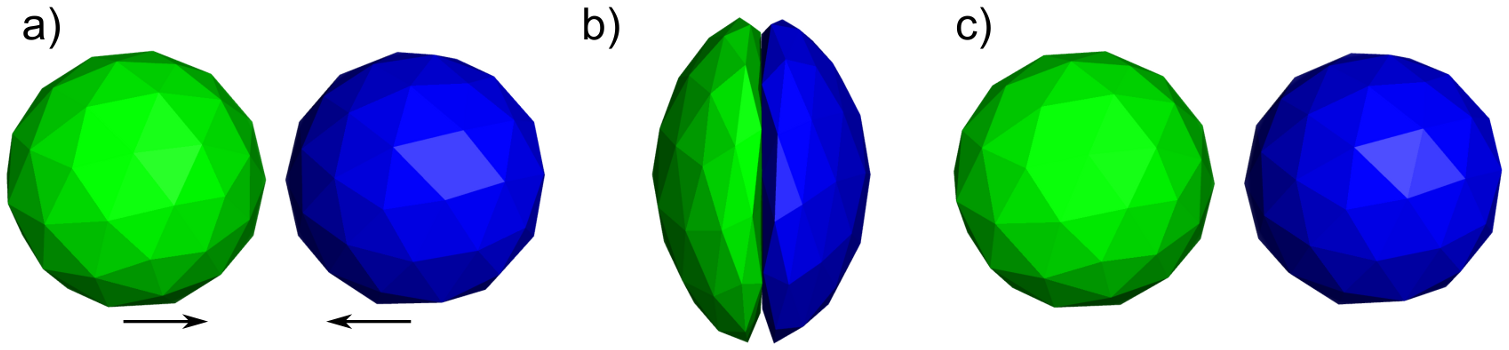
a) The two soft, viscoelastic spheres before collision. b) The spheres collide and suffer a large deformation. c) The spheres bounce back as a result of the steric interactions, recover their shape, and experience negligible rotation or deflection. A movie is available in the supplementary S3 File.

To test the Langevin scheme, we show that our implementation of both the springs and the steric forces are conservative, as required. The test performs an FFEA simulation in which two soft, viscoelastic spheres collide, as shown in Fig 19. As a result of the collision, the spheres undergo a large deformation before rebounding, losing energy as a result of dissipation, while still recovering their equilibrium shapes. The deflection of the spheres following the collision from their initial directions of travel prior to impact is small, and the largest rotation angle of a sphere about its own centroid is less than 3°.

## Availability and future directions

With this first release of the FFEA software, we aim to provide a multi-purpose simulation tool for the biomolecular modelling community to perform dynamic simulations of large protein assemblies based on low resolution structural information, such as is available in the EMDB. The software is released with a user-manual and tutorials, along with a test suite to validate every local install of the program. Planned future developments include improvements to the physics, such as inclusion of long-range hydrodynamics. To broaden the capabilities of FFEA, we are currently implementing the ability to switch between pre-defined protein conformational states, so that we can represent the power-stroke of molecular motors, for example. Moreover, we are introducing rod and sheet elements, in addition to tetrahedra, to facilitate FFEA modelling of one-dimensional objects such as coiled-coils, and two dimensional surfaces such as membranes. These additional capabilities will become available in subsequent software releases. There is also the potential to significantly improve code performance, such as through MPI parallelisation or porting to GPUs. Currently, a typical FFEA simulation running for several days on a desktop linux workstation will contain up to 10 interacting proteins, and will routinely explore *μ*s timescales. In the future, when the software can make use of multiple (e.g 1000) cores or accelerator technologies, we anticipate that far larger systems sizes (e.g 1000 interacting proteins) and longer timescales (up to ms) will be accessible. Given the success that MD simulations at the atomistic level have had in improving our understanding of the biomolecular structures within the PDB, new computational tools that bear an equivalent relationship to the EMDB have the potential to be extremely useful in interpreting the new experimental data from the biological mesoscale. FFEA is released under GPL license, made publicly available at https://bitbucket.org/ffea/ffea, documentation built automatically and stored at http://ffea.readthedocs.io/en/stable.

## Supporting information

S1 File Detailed physical and mathematical description of FFEA. This appendix details the physics and mathematics that FFEA model uses, and that have been implemented in the software package.
(PDF)

S2 File PCA comparison of atomistic and FFEA dynamics. Movie showing the highest overlapping mode for Arfaptin using an atomistic and FFEA trajectories (mapped back to atomistic coordinates).
(AVI)

S3 File Movie of two soft viscoelastic spheres colliding within FFEA. (AVI)

S1 Text Study of the flexibility of GroEL through FFEA simulation and analysis. The input scripts, structural information, output trajectories, measurement, and results for the GroEL simulations presented in this paper are made available at https://doi.org/10.5518/209 for FFEA version 2.4.
(DOCX)

S2 Text Analysis and comparison of FFEA with all-atom molecular dynamics. The input scripts, structural information, output trajectories, measurement, and results for the simulations on Arfaptin and xylanase, comparing FFEA and all-atom molecular dynamics presented in this paper, are made available at https://doi.org/10.5518/318.
(DOCX)
