# Supplementary material for: Fluctuating Finite Element Analysis (FFEA): A continuum mechanics software tool for mesoscale simulation of biomolecules
Source: PLoS Comput Biol. 2018 Mar 23;14(3):e1005897. doi: 10.1371/journal.pcbi.1005897 (PMC5891030; doi:10.1371/journal.pcbi.1005897)
Supplement: S1 File — This appendix details the physics and mathematics that FFEA model uses, and that have been implemented in the software package. (PDF) [file pcbi.1005897.s001.pdf]

Fluctuating Finite Element Analysis (FFEA): A Continuum Mechanics Software Tool for Mesoscale Simulation of Biomolecules. Supplementary Information.

Albert Solernou[1] Benjamin S. Hanson[1] Robin A. Richardson[2] Robert Welch[1] Daniel J. Read[3] Oliver G. Harlen[3] Sarah A. Harris[1]

1 School of Physics and Astronomy, University of Leeds, United Kingdom
2 School of Chemistry, University College London, United Kingdom
3 School of Mathematics, University of Leeds, United Kingdom

Details on the Finite Element decomposition in FFEA

For a system of interacting viscoelastic bodies subject to thermal fluctuations, the equation of motion within each of these bodies is given by the Cauchy momentum equation:

$$\rho \frac{D\boldsymbol{u}}{Dt} = \nabla \cdot (\boldsymbol{\sigma}^v + \boldsymbol{\sigma}^e + \boldsymbol{\pi}) + \boldsymbol{f} \tag{1}$$

where ρ is the density and $\frac{D\boldsymbol{u}}{Dt} = \frac{\partial \boldsymbol{u}}{\partial t} + \boldsymbol{u} \cdot \nabla \boldsymbol{u}$ is the Lagrangian (or material frame) derivative of the velocity with respect to time. Here $\boldsymbol{\sigma}^v$ the viscous stress, $\boldsymbol{\sigma}^e$ the elastic stress, $\boldsymbol{\pi}$ the thermal stress, and f the external force density all result from interactions with the rest of the system, such as internal thermal fluctuations, external fields and hydrodynamic drag against the solvent.

Oliver *et al.* [1] showed that Eq. 1 could be discretised using the finite element method [2] in a manner that allows the thermal stress to be determined locally. The weak form of Eq. 1 is obtained by integration over the volume of the body, Ω, with one of a set of weight functions, ω. Using index notation, this gives:

$$\int_\Omega \omega \left(\rho \frac{Du_i}{Dt} - \frac{\partial \sigma_{ij}}{\partial x_j} - f_i \right) dV = 0 \tag{2}$$

where the summation convention has been applied. Here the indices i and j refer to spatial directions and σ_{ij} is the total stress tensor. Applying integration by parts to the term involving the stress, gives

$$\int_\Omega \omega \frac{\partial \sigma_{ij}}{\partial x_j} = \int_\Gamma \omega F_i dA - \int_\Omega \sigma_{ij} \frac{\partial \omega}{\partial x_j} dV \tag{3}$$

where Γ is the surface of the body and $F_i = \sigma_{ij} n_j$ is the surface force density. We can therefore write the completed weak form of Eq. 1 as:

$$\int_\Omega \omega \rho \frac{Du_i}{Dt} + \int_\Omega \sigma_{ij} \frac{\partial \omega}{\partial x_j} dV = \int_\Omega \omega f_i dV + \int_\Gamma \omega F_i dA \tag{4}$$

By transferring the derivative to ω we require only continuity of the stress itself, and not its derivative. We now seek an approximate solution to Eq. 1 by finding a form for the velocity $\boldsymbol{u}$ from a restricted space of functions that satisfies Eq. 4 for a finite set of

weight functions ω. In the finite element method the space of functions is defined by dividing the integration domain into a set of 'finite elements' over which functions are restricted in form to piecewise polynomials. In our case, the finite elements are tetrahedra fixed in the Lagrangian frame of the material over which the velocity is restricted to being a linear interpolation of its values at the vertices (or nodes) of the tetrahedra. Consequently the value of the velocity at any point in the solution domain is entirely determined by its values at the nodes as $u_i(\boldsymbol{x}) = \sum_\alpha v_{i\alpha}\psi_\alpha(\boldsymbol{x})$, where $\psi_\alpha(\boldsymbol{x})$ are the linear interpolation functions, or *shape functions* and $v_{i\alpha}$ is the value of the ith component of velocity at node α. Furthermore, gradients of velocity are obtained from gradients of the shape functions $\psi_\alpha(\boldsymbol{x})$. The remaining task is to choose suitable weight functions ω. Here we use the Galerkin formulation in which ω are chosen to be the shape functions themselves.

The integrals in Eq. 4 can now be computed by adding up the contributions from each finite element to give a matrix equation of the form:

$$M_{pq}\frac{dv_q}{dt} = -\Lambda_{pq}v_q + E_p + N_p + O_p \tag{5}$$

where indices $p = i, \alpha$ and $q = j, \beta$ run over both basis functions, α, and Cartesian directions, i. M_{pq} is the mass matrix, Λ_{pq} is the viscosity matrix, E_p the elastic force vector, N_p the thermal force vector, and O_p the force vector arising from the external interactions.

The mass matrix, M_{pq}, has the form:

$$M_{p(i,\beta)q(j,\alpha)} = \delta_{ij}\left(\int_\Omega \rho\psi_\alpha\psi_\beta dV\right), \tag{6}$$

which has off-diagonal components which couple together nodes which share a finite element. However, since the nodes are embedded in the material and no mass is transported between the elements of a proteins, this matrix remains constant over the course of a simulation.

The matrix Λ_{pq} arises from the internal viscous stress and the hydrodynamic drag from the solvent background. The viscous stress is assumed to be isotropic and linear so that it takes the form:

$$\sigma^v_{ij} = \mu\left(\frac{\partial u_i}{\partial x_j} + \frac{\partial u_j}{\partial x_i}\right) + \lambda\frac{\partial u_m}{\partial x_m}\delta_{ij} \tag{7}$$

where μ is shear viscosity, and λ the second co-efficient of viscosity [3], giving a contribution to the viscosity matrix, Λ_{pq}, of

$$\begin{aligned}\Lambda^{\text{int}}_{p(i,\beta)q(j,\alpha)} &= \int_\Omega \frac{\partial\psi_\beta}{\partial x_j}\sigma^v_{ij}dV \\ &= \int_\Omega \mu\frac{\partial\psi_\beta}{\partial x_c}\frac{\partial\psi_\alpha}{\partial x_c}\delta_{ij} + \mu\frac{\partial\psi_\beta}{\partial x_j}\frac{\partial\psi_\alpha}{\partial x_i} + \lambda\frac{\partial\psi_\beta}{\partial x_i}\frac{\partial\psi_\alpha}{\partial x_j}dV\end{aligned} \tag{8}$$

FFEA represents interactions with the external solvent through a Stokes drag, the same as in Brownian dynamics simulations [4]. Each node experiences a local drag force that contributes an additional contribution

$$\Lambda^e_{pq} = -6\pi R_p\mu^s\delta_{pq} \tag{9}$$

to the viscosity matrix, where R_p is the effective radius of the node p and μ^s is the external solvent viscosity.

E_p, the elastic force vector, can be calculated from an associated elastic stress, σ^e_{ij}:

$$E_{p(i,\beta)} = -\int_\Omega \frac{\partial \psi_\beta}{\partial x_j} \sigma^e_{ij} dV \tag{10}$$

We assumed the bodies to be hyperelastic, and thus σ^e_{ij} can be calculated from a strain energy density functional [5], which results in:

$$\sigma^e_{ij} = G\frac{V_0}{V}\left(F^T_{ik}F_{kj} - \delta_{ij}\right) + \frac{1}{2}\left(\mathcal{K} - \frac{2G}{3}\right)\left(\frac{V}{V_0} - \frac{V_0}{V}\right)\delta_{ij} \tag{11}$$

where G and $\mathcal{K}$ are the shear and bulk modulus respectively, defined by the user. Here F_{ij} is the deformation gradient tensor ($F_{ij} = \frac{\partial x_i}{\partial X_j}$, where $\vec{x} = \vec{x}(\vec{X}, t)$ is the current position of the material initially located at $\vec{X}$), and V and V_0 are the current and initial element volumes, related by $V/V_0 = det(\mathbf{F})$.

The thermal force vector N_p must be chosen so that Eq. 5 satisfies the fluctuation-dissipation theorem. N_p must therefore be directly related to the form of the viscosity matrix Λ_{pq}. In general this would require computing the square-root of the viscosity matrix, which would be computationally intensive. However, one of the advantages of the FFEA formulation is that N_p can determined from only the local contributions from each element and each node. Since the internal viscous stresses arise from velocity gradients that are independent of the translation and rotation of the element they are entirely decoupled from the solvent drag terms that act on each element and so their contributions to N_p can be computed separately.

Since the velocity gradient is constant within each element, Oliver *et al.* [1] showed that the thermal stress within an element of volume V for a simulation timestep Δt is given by

$$\sigma^t_{ij} = \sqrt{\frac{2k_BT}{V\Delta t}}(X_{ij}\sqrt{\mu} + \delta_{ij}X_0\sqrt{\lambda}) \tag{12}$$

where X_0 an independent stochastic variable and X_{ij} a symmetric stochastic tensor, satisfying

$$\begin{aligned} \langle X_{ij}\rangle &= \langle X_0\rangle = 0 \\ \langle X_0 X_0\rangle &= 1 \\ \langle X_0 X_{ij}\rangle &= 0 \\ \langle X_{ij}X_{kl}\rangle &= \delta_{ik}\delta_{jl} + \delta_{il}\delta_{jk} \end{aligned}$$

from which the internal viscosity contribution to N_p can be assembled from

$$N^{\text{int}}_{p(i,\beta)} = -\int_\Omega \frac{\partial \psi_\beta}{\partial x_j}\sigma^t_{ij}dV. \tag{13}$$

Similarly, the dissipation due to Stokes drag against a solvent background gives an additional thermal contribution, N^e_p:

$$N^e_p = \left(\frac{12k_BT\pi R_p\mu^s}{\Delta t}\right)^{\frac{1}{2}} X^e_p \tag{14}$$

where X^e_p is an independent stochastic vector with the statistical properties $\langle X^e_p\rangle = 0$ and $\langle X^e_p X^e_q\rangle = \delta_{pq}$.

Finally, O_p is the force vector formed from the external interactions, which are discussed in the following sections.

Finite Element discretisation for the Lennard-Jones interactions

Lennard-Jones (LJ) interactions are often used for biomolecular modelling and more generally in soft matter simulations. We have extended this popular potential to a continuum implementation as a surface-surface interaction. In this representation, the force exerted on a surface point $\boldsymbol{s}$ by a surface Γ_t can be written as:

$$\boldsymbol{F}(\boldsymbol{s}) = \int_{\Gamma_t} \boldsymbol{f}(\boldsymbol{s},\boldsymbol{t}) dA_t \tag{15}$$

where $\boldsymbol{t}$ is a point on the surface Γ_t. The term $f(\boldsymbol{s},\boldsymbol{t})$ is consistent with the Lennard-Jones form:

$$\boldsymbol{f}(\boldsymbol{s},\boldsymbol{t}) = \frac{12\epsilon}{r^{eq}} \left[\left(\frac{r^{eq}}{r(\boldsymbol{s},\boldsymbol{t})} \right)^{13} - \left(\frac{r^{eq}}{r(\boldsymbol{s},\boldsymbol{t})} \right)^{7} \right] \hat{\boldsymbol{r}} \tag{16}$$

where $\boldsymbol{r} = \boldsymbol{s} - \boldsymbol{t}$ is the vector displacement between the points $\boldsymbol{s}$ and $\boldsymbol{t}$ and $r(\boldsymbol{s},\boldsymbol{t}) = |\boldsymbol{r}|$ the corresponding distance between the points. The parameters controlling this interaction are r^{eq}, the equilibrium distance for the LJ interaction, and ϵ, the energy minimum at $r(\boldsymbol{s},\boldsymbol{t}) = r^{eq}$. Including this interaction in FFEA means introducing Eq. 15 into the RHS of Eq. 4:

$$\int_{\Gamma_s} \omega(\boldsymbol{s}) \boldsymbol{F}(\boldsymbol{s}) dA_s = \int_{\Gamma_s} \omega(\boldsymbol{s}) \left[\int_{\Gamma_t} \boldsymbol{f}(\boldsymbol{s},\boldsymbol{t}) dA_t \right] dA_s. \tag{17}$$

Hence the contribution to O_p from the Lennard Jones interactions is given by:

$$O_p^{LJ} = \int_{\Gamma_s} \psi_\alpha(\boldsymbol{s}) \left[\int_{\Gamma_t} f_i(\boldsymbol{s},\boldsymbol{t}) dA_t \right] dA_s. \tag{18}$$

where the weight functions $\omega(\boldsymbol{s})$ have been replaced with the surface shape functions $\psi_\alpha(\boldsymbol{s})$ as per the Galerkin formulation of finite element analysis, and $p = \alpha, i$ is the index for the co-ordinate referring to node α in direction i. This double integral can then be partitioned into a double sum of integrals over all pairs of triangular surface faces S and T, written as

$$O_p^{LJ} = \sum_S \sum_T \int_S \int_T \psi_\alpha(\boldsymbol{s}) f_i(\boldsymbol{s},\boldsymbol{t}) dA_T dA_S, \tag{19}$$

Now, we use a Gaussian quadrature scheme for triangles to approximate each integral as a weighted sum:

$$\int_S g(\boldsymbol{s}) dA_S \sim A_S \sum_k^{N_G} W_k g(\boldsymbol{s}_k) \tag{20}$$

where the weighted sum is of the integrand's values at N_G of the Gauss Points, $\boldsymbol{s}_k$, and A_S is the area of the triangular element S. We use Gaussian quadrature of degree two for which $N_G = 3$. Using this scheme to evaluate Eq. 19, allows us to construct O_p^{LJ} as

$$O_p^{LJ} = \sum_S \sum_T A_S A_T \sum_{k=1}^{N_G} \sum_{l=1}^{N_G} W_k W_l \psi_\alpha(\boldsymbol{s}_k) f_i(\boldsymbol{s}_k, \boldsymbol{t}_l). \tag{21}$$

It should be noted that this sum only contributes to nodes α that are on the surface of the finite element mesh, and that the sum over S only has non-zero contributions from elements containing the node α. It is also true that the net interaction of a face with itself is zero. Since the force decays rapidly with distance, we impose a cut-off distance and only consider pairs of faces where the minimum distance is below the cut-off length.

Linear Elastic Model & Timescale Calculator

The linear elastic model is determined by a manual linearisation of the elasticity vector E_p about the equilibrium structure $\boldsymbol{x}^0$:

$$E_p \approx E_p|_{\boldsymbol{x}^0} + \frac{\partial E_p}{\partial x_q}\left(x_q - x_q^0\right) \tag{22}$$

where we define the spring constant matrix $K_{pq} = \frac{\partial E_p}{\partial x_q}$. Due to the complex form of E_p following the finite element method, we calculate its partial derivative numerically, i.e. $\frac{\partial E_p}{\partial x_q} \approx \frac{\Delta E_p}{\Delta x_q}$. Following initialisation of the system in which all material parameters are assigned to the object, we loop over all of the nodes, β, and move each a small distance Δx in each of the three directions, j. Following this small deformation, we calculate the total elastic force vector E_p using algorithms from the core FFEA algorithm. This allows us to determine how each component of ΔE_p changes with a deformation Δx_q, where the index $q = \beta, j$. Looping over the entire structure of N nodes gives us a $3N \times 3N$ matrix, K_{pq}. This matrix is symmetric and invertible if the distance Δx_q is kept sufficiently small, which is expected for an effective elastic network matrix [6]. We calculate an appropriate Δx_q as:

$$\Delta x_q = \frac{1}{100}\sqrt[3]{v_s} \tag{23}$$

where v_s is the volume of the smallest element in the system. This is approximately 1% of the smallest length-scale associated with the entire system, and is kept the same for all indices q.

Diagonalisation of K_{pq} gives us the following:

$$\mathbf{K} = \mathbf{V}\mathbf{k}\mathbf{V}^{-1} \tag{24}$$

where $\mathbf{V}$ is the matrix of eigenvectors, arranged as columns, which represent the elastic normal modes of motion and $\mathbf{k}$ is the diagonal matrix of eigenvalues, each representing the stiffness of their respective elastic mode. The equipartition theorem allows us to transform from stiffnesses to actual motions using the covariance matrix $C_{pq} = \langle x_p x_q \rangle$. As FFEA follows a Boltzmann distribution for energies, it can be shown that:

$$C_{pq} = k_B T K_{pq}^{-1} \tag{25}$$

showing that the eigenvectors of C_{pq} are the same as those of K_{pq}, with the eigenvalues differing only by a factor of $k_B T$. It follows that the square root of these new eigenvalues gives us a characteristic deformation lengthscale to associate with each elastic mode. Using this together with the associated eigenvector, we can generate a small pseudo-trajectory of 20 frames by moving the each node in the system according to a normalised eigenvector multiplied by this characteristic lengthscale, allowing a visualisation of the types of motion available to the system.

We can also follow a similar technique to find the timescales associated with each elastic mode of the system, as well as the ballistic modes associated with the mass matrix, M_{pq}. Taking only the inertial and viscosity terms of Eq. 5, we obtain:

$$M_{pq}\frac{\partial v_q}{\partial t} = -\Lambda_{pq} v_q \tag{26}$$

which rearranges to:

$$\tau_{pq}^m \frac{\partial v_q}{\partial t} = -v_q \tag{27}$$

where $\tau^m_{pq} = \Lambda^{-1}_{pr} M_{rq}$. Diagonalisation of this matrix gives a set of eigenmodes, with a spectrum of relaxation times as the eigenvalues. These eigenvalues give an indication of the time required for relaxation of inertia within the simulation.

Similarly, we can take only the viscosity and linearised elastic terms of Eq. 5, which gives us:

$$\Lambda_{pq} \frac{\partial x_q}{\partial t} = -K_{pq} x_q \tag{28}$$

and rearranges to:

$$\tau^k_{pq} \frac{\partial x_q}{\partial t} = -x_q \tag{29}$$

where $\tau^k_{pq} = K^{-1}_{pr} \Lambda_{rq}$. The previous decomposition approach provides a new set of eigenvalues, this time corresponding to elastic relaxation times within the simulation.

Note that the eigenmodes corresponding to the inertial and elastic relaxation times are different from one other and from the eigenmodes calculated using the linear elastic model, and that the elastic modes of motion do not necessarily corresponded to the actual dynamical modes of motion emerging from a full simulation. It should also be noted that the motion may be underdamped or overdamped, leading to mixing of timescales for simulations using the inertial scheme with Eq. 5 as the equation of motion. We therefore emphasise that the linear elastic model, and these timescales, are an approximation.

Nevertheless, the timescales emerging from these two matrices present useful information. The largest timescale within the spectra tells us approximately how long our simulation must be to capture every type of motion available to the biomolecule in question. For a statistically complete ensemble of motions, the simulation time, t_s, must be hundreds of times longer than the longest of these timescales. The smallest timescale in the system tells us how often we must output trajectory data in order to capture the fastest motion within the molecule, and therefore how short our simulation timestep, Δt, must be. In fact, for an FFEA simulation to be numerically stable, Δt *must* be smaller than the smallest of these eigenvalues, as the equations of motion are solved explicitly.

References

1. Oliver R, Read DJ, Harlen OG, Harris SA. A Stochastic Finite Element Model for the Dynamics of Globular Macromolecules. J Comp Phys. 2013;239:147--65.

2. Bathe KJ. Finite element method. Wiley Online Library; 2008.

3. Tritton DJ. Physical fluid dynamics. Springer Science & Business Media; 2012.

4. Leimkuhler B, Matthews C. Molecular dynamics: with deterministic and stochastic Numerical Methods. Interdisciplinary Applied Mathematics. 2015;39.

5. Shontz SM, Vavasis SA. A robust solution procedure for hyperelastic solids with large boundary deformation. Engineering with Computers. 2011;28(2):135--47.

6. Lezon TR, Shrivastava IH, Yang Z, Bahar I. Handbook on Biological Networks (Ch. 7). 57 Shelton Street, Covent Garden, London, WC2H 9HE: World Scientific Publishing Co. Pte. Ltd.; 2010.
