# Supplementary material for: Fluctuating Finite Element Analysis (FFEA): A continuum mechanics software tool for mesoscale simulation of biomolecules
Source: PLoS Comput Biol. 2018 Mar 23;14(3):e1005897. doi: 10.1371/journal.pcbi.1005897 (PMC5891030; doi:10.1371/journal.pcbi.1005897)
Supplement: S1 Text — The input scripts, structural information, output trajectories, measurement, and results for the GroEL simulations presented in this paper are made available at https://doi.org/10.5518/209 for FFEA version 2.4. (DOCX) [file pcbi.1005897.s004.docx]

S1 Text. Study of the flexibility of GroEL through FFEA simulation and analysis. The input scripts, structural information, output trajectories, measurement, and results for the GroEL simulations presented in this paper are made available at <https://doi.org/10.5518/209> for FFEA version 2.4.
