# Supplementary material for: Fluctuating Finite Element Analysis (FFEA): A continuum mechanics software tool for mesoscale simulation of biomolecules
Source: PLoS Comput Biol. 2018 Mar 23;14(3):e1005897. doi: 10.1371/journal.pcbi.1005897 (PMC5891030; doi:10.1371/journal.pcbi.1005897)
Supplement: S2 Text — The input scripts, structural information, output trajectories, measurement, and results for the simulations on Arfaptin and xylanase, comparing FFEA and all-atom molecular dynamics presented in this paper, are made available at https://doi.org/10.5518/318. (DOCX) [file pcbi.1005897.s005.docx]

S2 Text. Analysis and comparison of FFEA with all-atom molecular dynamics. The input scripts, structural information, output trajectories, measurement, and results for the simulations on Arfaptin and xylanase, comparing FFEA and all-atom molecular dynamics presented in this paper, are made available at <https://doi.org/10.5518/318>.
